# Meldrum’s acid assisted formation of tetrahydroquinolin-2-one derivatives a short synthetic pathway to the biologically useful scaffold

**DOI:** 10.1038/s41598-023-50535-0

**Published:** 2024-01-04

**Authors:** Małgorzata Ryczkowska, Alicja Trocka, Anna Hromova, Sławomir Makowiec

**Affiliations:** Department of Organic Chemistry, Faculty of Chemistry, Gdansk University of Technology, Narutowicza 11/12, 80-233 Gdańsk, Poland

**Keywords:** Organic chemistry, Drug development

## Abstract

A new method for the preparation of tetrahydroquinolin-2-one derivatives is presented. This approach involves a two-step reaction between enaminones and acylating agents, immediately followed by electrophilic cyclization, all within a single synthesis procedure, eliminating the need to isolate intermediates. The entire process is facilitated by the use of acyl Meldrum’s acids which not only shortens the preparation time of the substrates but also easily extends the range of substituents That can be used. The method’s scope and limitations were evaluated with various reagent combinations thus demonstrating its general applicability to the synthesis of tetrahydroquinolin-2-one core. Interestingly, some exceptions to the regular reaction pathway were observed when a strong EDG (electron donating group) was introduced via acyl Meldrum’s acids. The underlying mechanism of this phenomenon was elucidated during the investigation.

## Introduction

**Figure 1:**
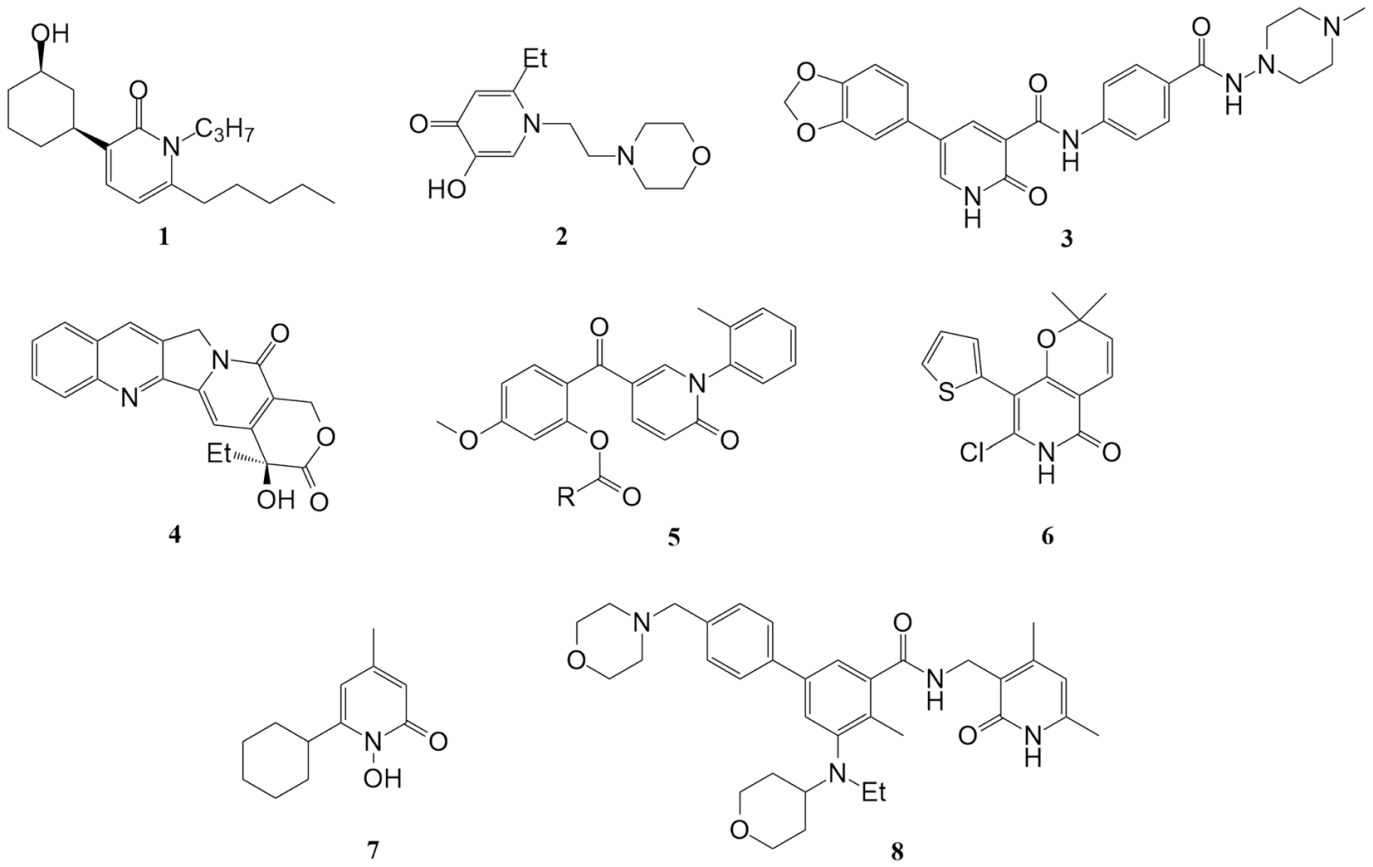
Examples of bioactive derivatives containing pyridone moiety.

The pyridone motif is commonly found in a diverse range of biologically active compounds, including pharmaceuticals, with a primary focus on the drug-like properties of pyridone derivatives. Consequently, pyridine molecules (Fig. 1) offer a wide range of potential biological targets. These include, for example, agonists of the cannabinoid CB2R receptor **1**,^1,2^ analgesic agents featuring the 4-pyridone moiety **2**^3,4^ kinase inhibitors **3** with anaplastic anticancer properties^5^, and the topoisomerase inhibitor camptothecin **4**. Additionally, anticancer and antiproliferative activities are observed in 2-pyridone compounds, as reported by Lvi and colleagues **5**^6^. Polycyclic pyridones **6** also exhibit antifungal activity against *Candida albicans*^7^. Notably. There are numerous commercially available drugs among pyridone derivatives, such as the antifungal and antibacterial agent ciclopirox **7**, and the antisarcoma drug tazemetostat **8**.

On the other hand, the use of pyridines as biologically active agents comes with an additional advantage related to their rapid biodegradation^8,9^.

**Figure 2:**
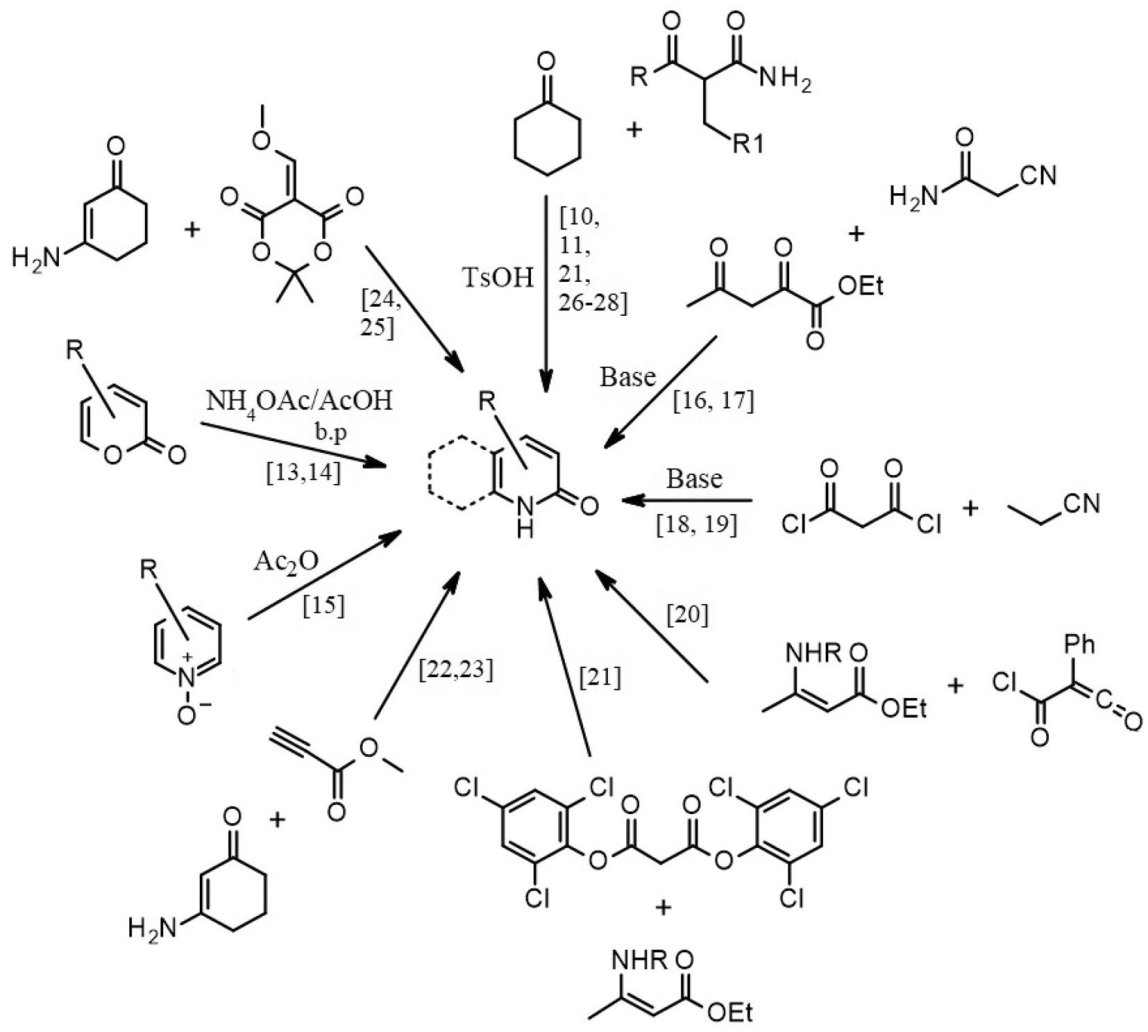
Selected examples of the synthesis routes of 2-pyridones.

Our recent synthetic efforts have been directed towards the synthesis of 2-pyridone moieties fused with saturated rings, as well as the preparation of permanently aromatic systems that are isosteric with the pyridone scaffold^10,11^. As per our expectations these compounds have exhibited, according to the molecular docking results, anticancer activity, although the synthesis process had room for improvement. The chemical literature provides a significant number of synthetic methods for preparing the 2-pyridone moiety. Thus, from the most popular methods, we can distinguish two main approaches^12^. The first approach involves transforming existing heterocyclic rings into 2-pyridone and the second is based on the formation of the heterocyclic ring de novo. According to the first approach, pyrones could be easily transformed into pyridone rings by heating pyrones in boiling acetic acid in the presence of ammonium acetate^13,14^ (Fig. 2). Alternatively, pyridine N-oxides can be transformed into pyridines by heating with carboxylic acid anhydrides^15^.

The second approach involves forming the ring from non-cyclic starting materials, frequently through the condensation of a nitrogen-bearing component with 1,3-dicarbonyl compounds. An example is the reaction of cyanoacetamide with ethyl 2,4-dioxovalerate^16,17^. Additionally, nitriles can serve as a source of nitrogen when reacting with highly reactive malonyl chloride^18,19^. A unique and interesting type of condensation to form 2-pyridones involves reactions where the nitrogen component takes the form of an enamine, reacting with 1,3 dicarbonyl derivatives. Sheibani and co-workers published a paper where they described the reaction of an enamine conjugated with carbonyl reacts with (chlorocarbonyl)phenyl ketene^20^. A somewhat similar approach was used by Guillemont and co-workers, who detailed the formation of the 2-pyridone scaffold in the reaction of a conjugated enamine with an activated malonic derivative^21^. A completely different approach assumes the application of methyl propiolate with enaminone^22,23^. The method using methoxymethylene Meldrum’s acid and enaminone also deserves mention^24,25^.

The method previously used in our laboratory for the synthesis of the tetrahydroquinolin-2-one scaffold^10,11^ was a combination and adaptation procedures described by several research groups^26–28^. However, due to difficulties with the direct procedure involving benzoyl acetate, ammonium acetate, and cyclohexanone, where the in-situ formation of an amide was expected, followed by subsequent reaction with cyclohexanone, we opted to synthesize the amide separately, isolate it, and then proceed to condense the 1,3-dicarbonyl amide with cyclohexanone to obtain the desired 2-pyridone fused with a six-membered ring. Although this method was successful, it proved to be tedious and time-consuming in practice, prompting our search for an improved method for the preparation of a 2-pyridone scaffold.

## Results and discussion

In this current paper, using our experience in synthesizing pyridones along with our knowledge about the applications of Meldrum’s acid^29–34^ in synthesis, and considering the available literature, we would like to introduce a new approach for the preparation of 2-pyridones.

**Figure 3:**
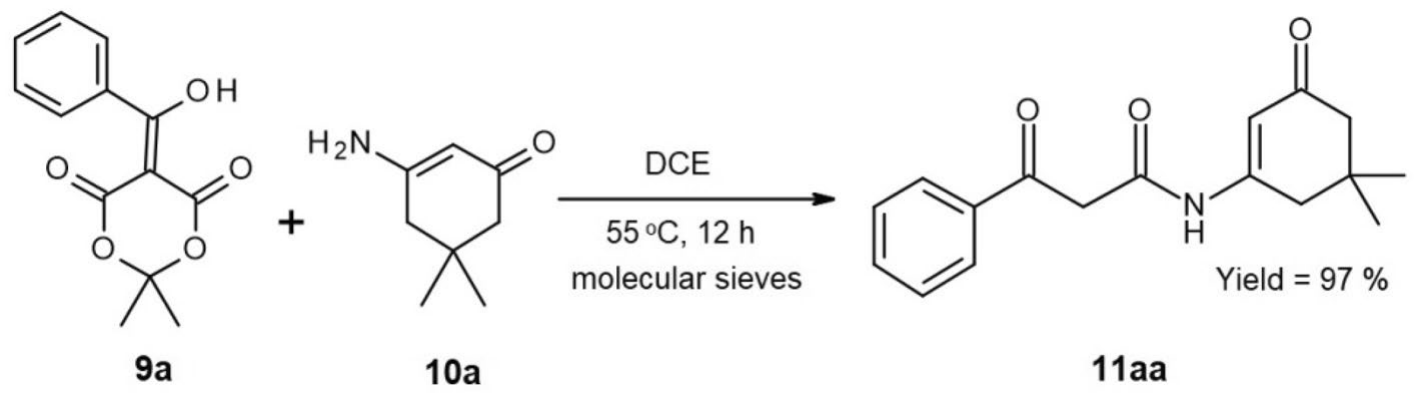
Preparation of enamide **11aa** with benzoyl Meldrum’s acid **9a**.

Taking into account our past experience, we identified a key step to be obtaining a 1,3-dicarbonyl amide with an already attached fragment that allows cyclization, resulting in a 2-pyridone fused with a hexagonal ring, like the tetrahydroquinolin-2-one. The enamide **11** in Fig. 3 aligns with these criteria. At this stage, we assumed that such an enamide would readily undergo the desired cyclization under acidic conditions.

In theory, obtaining the appropriate enamide should be straightforward with the enaminone and acylating reagent being available. However, using acyl-acetate esters is not recommended due to their low reactivity with amino groups and potential side reactions with the ketone fragment. However, the use of strong acylating reagents like chlorides, anhydrides, or ketenes, as seen in cited works^18–21^, presents challenges in preparation and storage. This led us to consider exploiting the acylating properties of Meldrum’s acid derivatives, which would allow us to introduce 1,3-dicarbonyl moiety together with the possibility of introducing a broad scope of side chains. It should be noted that the used acyl derivatives of Meldrum’s acid are stable compounds, easily purified and prepared from commercially available starting materials.

First, we conducted several experiments with 4 eq of 5-(hydroxy(phenyl)methylene)-2,2-dimethyl-1,3-dioxane-4,6-dione (**9a**) and 1 eq of 3-amino-5,5-dimethylcyclohex-2-enone (**10a**) under various conditions to optimize the method (Table S1 in SI). With these experiments, we observed the formation of the desired conjugated enamide, with yields ranging from a moderate 30% in the case of a reaction performed in boiling DCE (1,2-dichloroethane) without molecular sieves, up to a quantitative yield of 97% when molecular sieves were present, and the reaction was carried out at 55 °C in DCE (Fig. 3).

Having isolated and purified enamide **11aa**, we proceeded with its cyclization to form 7,7-dimethyl-4-phenyl-7,8-dihydroquinoline-2,5(1H,6H)-dione (**12aa**). Our initial attempts focused on the application of mild Lewis acids as transition metals triflates, especially scandium triflate. Unfortunately, this approach proved unsuccessful under various conditions.. This failure prompted us to search for an effective catalyst for the preparation of product **12aa**. We focused on protic acid catalysts, especially on polyphosphoric acid (PPA) known for its moderate acidity and its ability to catalyze similar reactions, including Knorr-type cyclization^35–41^.

**Figure 4:**
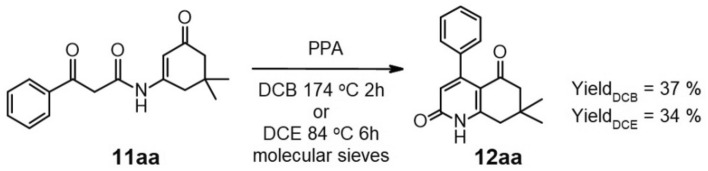
Formation of 7,7-dimethyl-4-phenyl-7,8-dihydroquinoline-2,5(1H,6H)-dione (**12aa**).

We conducted two experiments with the cyclization of enamide **11aa** in the presence of PPA. The first, carried out in boiling dichlorobenzene (DCB) for 2 h, resulted in the formation of compound **12aa** with a 37% yield, while the second was performed in boiling dichloroethane (DCE) within 6 h, yielding 34% (Fig. 4).

**Figure 5:**
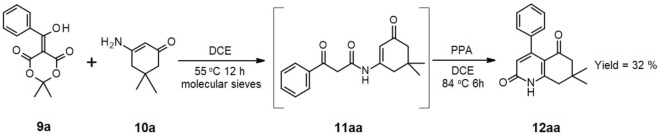
“One-pot” process for synthesis of derivative **12aa**.

With these outcomes, we explored the possibility of conducting both the intermolecular and intramolecular processes without the isolation of the enamide intermediate in a “one-pot” reaction. To test this hypothesis, we initiated the condensation of a fourfold excess of benzoyl Meldrum’s acid **9a** with enaminone **10a**. Once the enamide formation was complete and the disappearance of benzoyl Meldrum acid was confirmed through TLC analysis, PPA was introduced to the reaction, and the entire mixture was heated to its boiling point for 6 h. This resulted in the formation of product **12aa** with a yield of 32% (Fig. 5). As an alternative to the use of PPA we tested TsOH in toluene; however, this resulted in a weaker outcome since the yield after both steps combined was only 15%.

Encouraged by these findings, we decided to perform a series of reactions using various derivatives of Meldrum's acid and enaminones to evaluate the scope and limitations of this newly developed method. With our new method, we were able to prepare a wide range of compounds efficiently with a 7,8-dihydroquinoline-2,5(1H,6H)-dione scaffold in a single laboratory step, achieving moderate yields (Table 1).

**Table 1:** Synthesis of 7,8-dihydroquinoline-2,5(1H,6H)-dione derivatives from acyl Meldrum’s acids and enaminones.


Run	R^1^	R^2^	R^3^	Product	Yield [%]
1	Ph	CH_3_	CH_3_	12aa	32
2	3-CF_3_C_6_H_4_	CH_3_	CH_3_	12ba	29
3	4-CH_3_OC_6_H_4_	CH_3_	CH_3_	13ca^a^	24^a^
4	CH_3_	CH_3_	CH_3_	12da	33
5	Et	CH_3_	CH_3_	12ea	31
6	Ph	H	H	12ab	37
7	Ph	H	CH_3_	12ac	20
8	Ph	H	Ph	12ad	22
9	(1-Naph)-CH_2_	CH_3_	CH_3_	–^b^	–

^a^Structurally different product was obtained with 24% yield.

^b^Decomposition of the initially formed enamide **11fa.**

**Figure 6:**
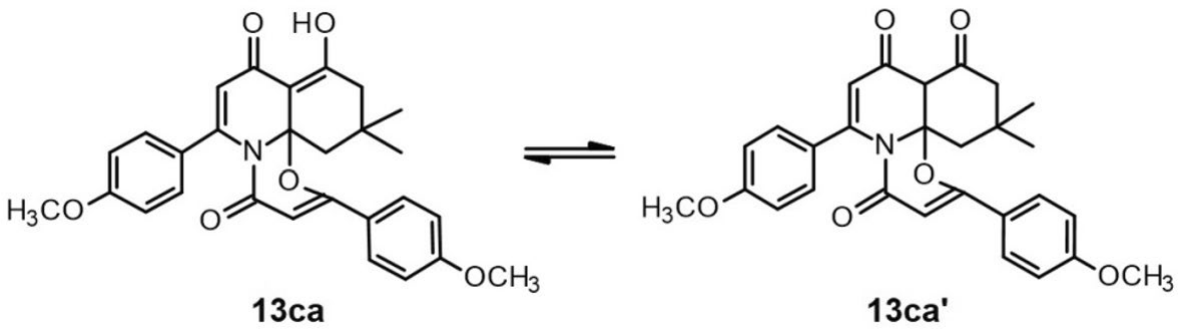
Proposed structure of unexpected cyclization product.

In the case of 5-(1-hydroxy-2-(naphthalen-1-yl)ethylidene)-2,2-dimethyl-1,3-dioxane-4,6-dione (**9f**) used as a Meldrum’s acid (Table 1, Run 9), enamide formation was observed but subsequent condensation using PPA was unsuccessful. A similar situation arose when 3-aminocyclopent-2-enone was applied. Surprisingly, when derivative **9c** was used as a Meldrum’s component (Table 1, Run 3), a notably different product compared to the other results was isolated from the reaction mixture. Determining the structure of this unexpected product was an essential step in our analysis. In became evident that this compound contained a double fragment originating from the Meldrum derivative and only one from the enaminone. Based on data from NMR and MS, we proposed the following structure, which may exist in equilibrium with its keto form (Fig. 6).

**Figure 7:**
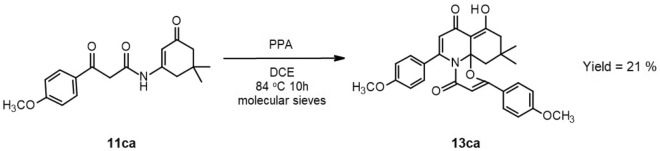
Formation of “unexpected” product during cyclization of enamide.

This prompted us to investigate the reaction mechanism behind this particular outcome. First, we sought to determine the step in the process that was responsible for the formation of this unusual product. Since the unexpected product contained an “excess” of moieties originating from acyl Meldrum’s acid, it suggested that the surplus of compound **9c** used in a “one-step” process might be causing this atypical reaction course. To test this, we prepared enamide **11ca** following the stepwise procedure, and isolated and purified it. In the next step, we attempted to cyclize purified **11ca** using PPA. This reaction again yielded compound **13ca** with a 21% yield (Fig. 7).

**Figure 8:**
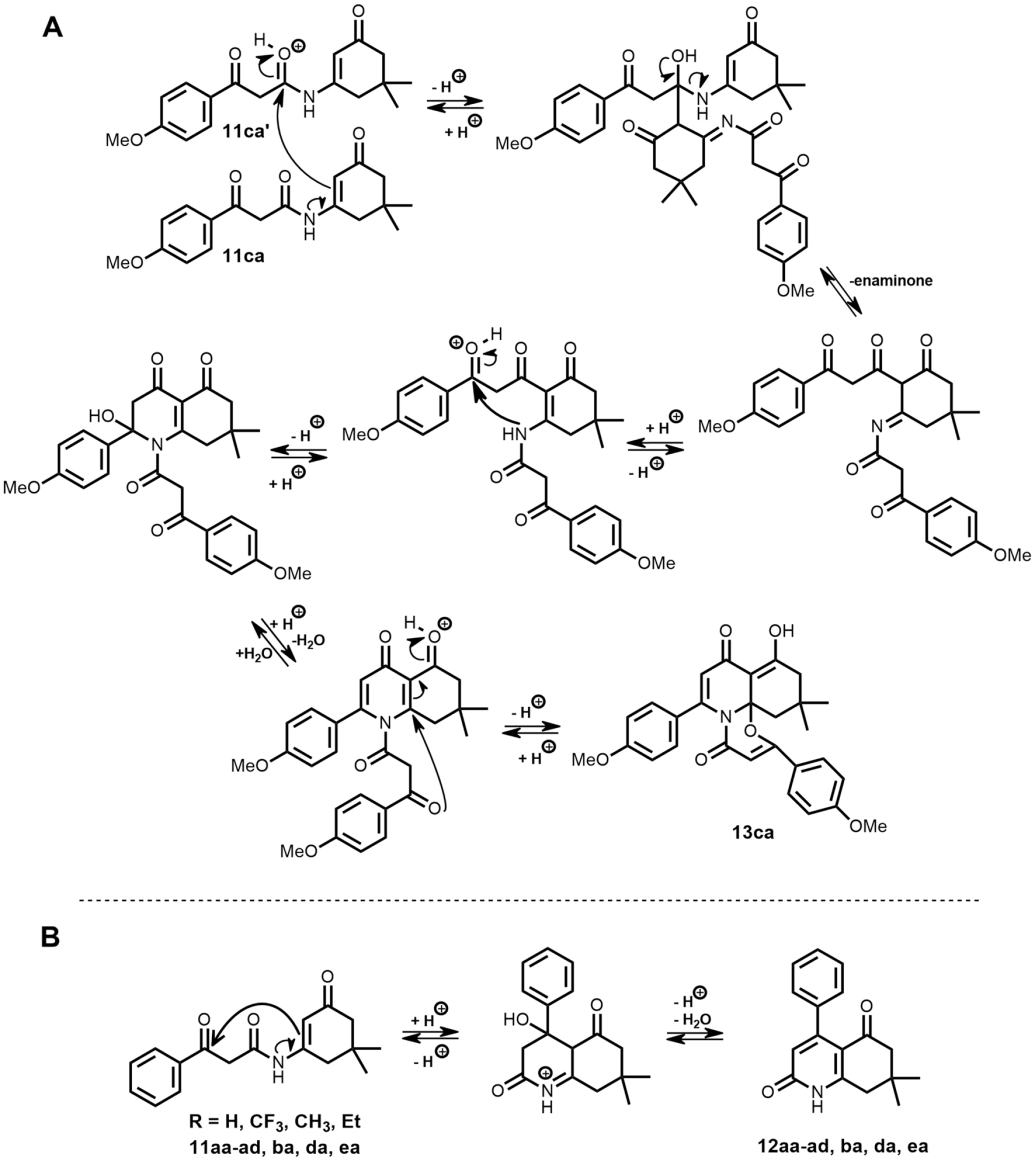
(**A**) Tentative reaction mechanism proposed for the intermolecular formation of **13ca**; (**B**) Competitive intramolecular reaction for compounds 11 with weak EDG or EWG.

The result confirmed that the excess of acyl Meldrum’s acid used in the “one-pot” reaction wasn’t responsible for the unusual course of the reaction. Compound **13ca** seemed to be formed through the interaction of two molecules of enamide **11ca**. To explain the formation of product **13ca** we proposed a tentative reaction mechanism presented in Fig. 8A.

**Figure 9:**
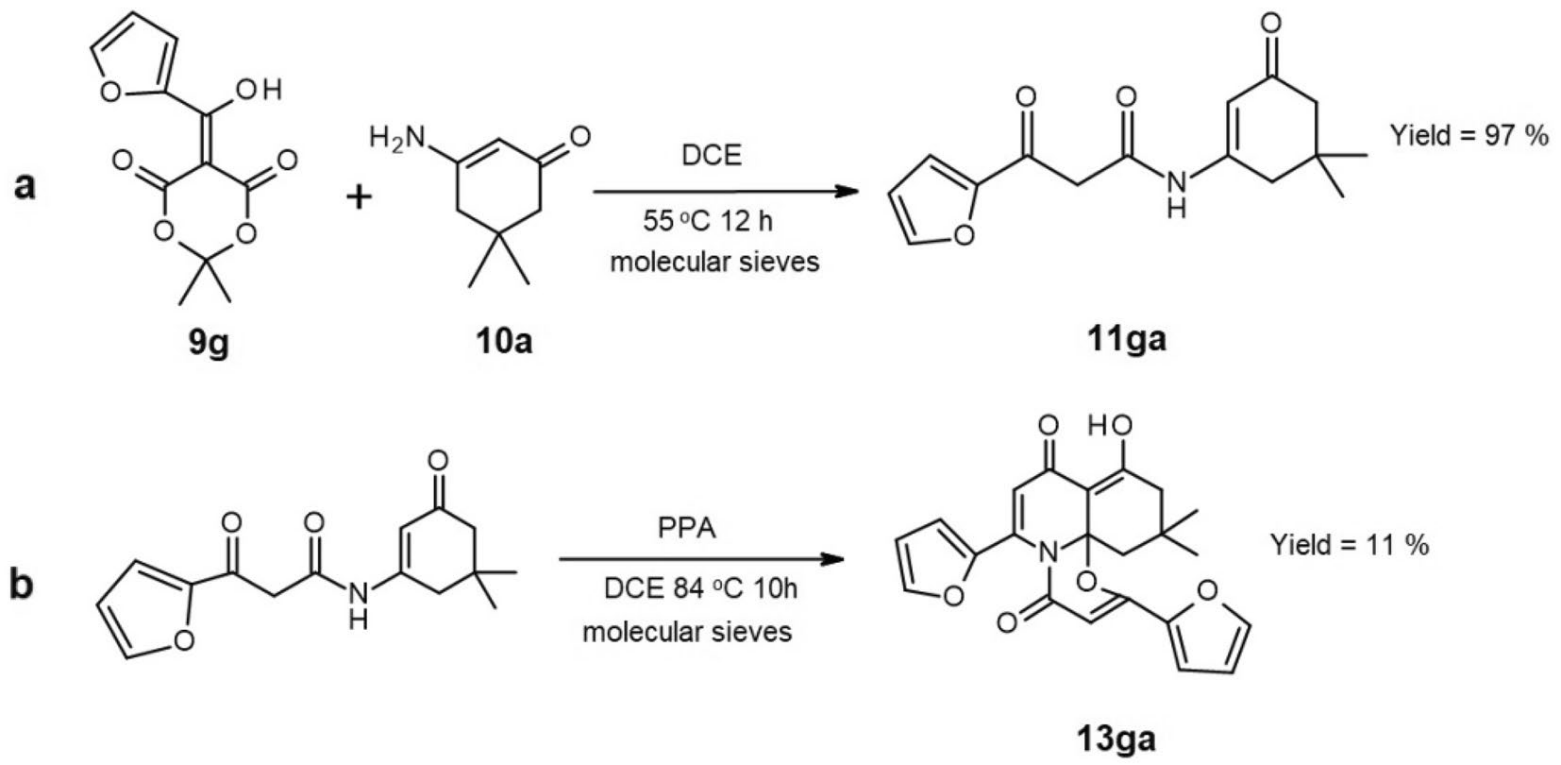
Reactions carried out to investigate the effect of the strong EDG on the course of condensation.

Obviously, we paid attention to the fact that only the reaction of *p*-methoxy derivative of Meldrum’s acid with dimedone enaminone gave us an unexpected product. Thus, to explain this phenomenon, we hypothesized that EDG caused a decrease in the electrophilicity of the keto carbonyl carbon in enamide **11ca**. This inhibited preferred intramolecular cyclization process (Fig. 8B) which would typically lead to the usual product **12ca** and simultaneously enabled the observation of a more entropically challenging intermolecular process. To validate our hypothesis, we decided to obtain and purify enamide **11ga** with a furyl substituent possessing a strong M+ effect (Fig. 9a). Subsequently, we carried out the intramolecular condensation with PPA once again, resulting in the formation of compound **13ga** which confirmed that an EDG indeed affects the course of the reaction (Fig. 9b).

In both intermolecular (Fig. 8A) and intramolecular (Fig. 8B) reactions, the enaminone fragment plays the role of the nucleophile, with the largest contribution to the HOMO orbital of the molecule. In the discussed intermolecular condensation, it is theoretically possible for the nucleophile to attack either the keto-carbonyl (not shown in Fig. 8A) or the amide carbonyl carbon atom. Considering the factors affecting the transition of the reaction from intramolecular to intermolecular (the presence of ED group decreases the electrophilicity of keto-carbonyl carbon atom) it is likely that the enaminone nucleophile initiates the reaction with the amide carbonyl, as otherwise, we would observe with intramolecular cyclization like in substrates **11aa**–**ad**, **ba**, **da**, **ea** when a weak EDG or EWG (electron withdrawing group) is present, which implies higher electrophilicity of keto-carbonyl than amide carbonyl carbon atom.

**Figure 10:**
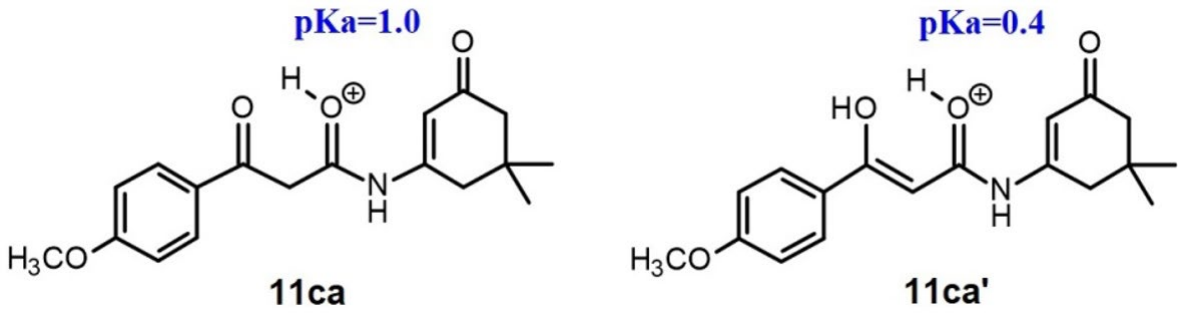
Conjugate acids to ketone and enol form of *N*-(5,5-dimethyl-3-oxo-cyclohexene-1-yl)-3-(4-methoxyphenyl)-3-oxo-propanamide.

Moreover, in our search for the most basic position of the molecule, we estimated p*K*a values for the conjugate acids of enamide **11ca** and its enol form **11ca**′ using the “Chemaxon p*K*a calculator”^42^. The calculated p*K*a values were 1.0 and 0.4, respectively (Fig. 10).

In an environment with an excess of PPA, these compounds will be predominantly in the protonated form. The protonated forms are more electrophilic than the non-protonated ones. When considering the participation of these two forms in the actual course of the reaction, the position of the keto-enol equilibrium of protonated and deprotonated forms should also be taken into account. Based on the ^1^H NMR spectra for EDG-substituted enamides in a non-polar solvent, the enol form was negligibly small. Consequently the most electrophilic species in our reaction mixture would be the protonated enamide **11ca** (Fig. 10), providing further support for our proposed mechanism.

## Conclusion

In this paper, we introduced a “one-pot” reaction developed by our team for the synthesis of 4-phenyltetrahydroquinolone cores. This process involves an initial formation of an appropriate enamide as an intermediate, followed by its condensation with PPA. The presented method has significant advantages over previously used procedures^26,27^. The “one-pot” synthesis allows obtaining the desired tetrahydroquinolone core within several hours, in mild conditions, without the intermediate isolation. The yields of the proposed synthesis are higher than those previously published (e.g. 12%^27^; 18–21% in two steps^43^; 16% in two steps, 27%^25^). The condensation of Meldrum's acid derivatives with enaminones also allows the tetrahydroquinolone core functionalization in the 4-position with alkyl substituents, which was not possible before due to the difficulties of ethyl acetoacetate and its alkyl derivatives ammonolysis. Additionally, the presence of carbonyl carbon in the 5-position opens new possibilities for tetrahydroquinolone core modification. Nevertheless, the presented method does have its limitations. The reaction predominantly proceeds through intramolecular cyclization for enamides that lack strong EDG. In contrast, for those with ED groups, we observed an intermolecular condensation pathway.

## Methods

### General

Commercially available reagents were purchased from Sigma Aldrich or Acros and used without further purification. Acyl Meldrum’s acids **9a**–**f** and enaminones **10a**–**d** were prepared according to literature procedures; **9a**, **9b**, **9c**, **9f**^44^, **9d**, **9e**^45^, **10a**–**d**^46^. Analytical thin layer chromatography was performed on aluminum sheets of UV 254 Merck silica gel, and flash chromatography using SilicaFlash P60 silica gel (40–63 µm). ^1^H and ^13^C NMR spectra were recorded with Bruker Avance III HD 400 MHz or Varian Gemini 500 MHz and NMR chemic al shifts were reported in δ (ppm) using residual solvent peaks as standards, with the coupling constant J measured in Hz. High resolution mass spectra were recorded with an Agilent 6540 Q TOF system High resolution (HRMS) was recorded on Agilent 6540 QTOF.

### General procedure for preparation of compounds 12aa-da, 13ga

A solution of enaminone **10a**–**d** (0.5 mmol) in 5 ml of DCE and molecular sieves were placed in a round bottom flask with a stir bar and heated to 55 °C. Then 2 mmol of acyl Meldrum's acid **9a**–**f** were added in 4 portions every 1 h. The formation of enamide was monitored by TLC. When the spot of enaminone was no longer observed 0.8 g of PPA was added. The reaction mixture was then heated to reflux, left for 6 h and after that DCE was evaporated. The residue was then suspended in water, cooled in the ice bath and neutralized with NaOH. Next, the resulting suspension was subjected to extraction with AcOEt and DCM. Organic layers were washed with brine and dried with anhydrous MgSO_4_ The final product was isolated by flash column chromatography (C:M 200:1 and if needed A:H 2:1).

### General procedure for preparation of compounds 11aa–ga

A solution of dimedone enaminone **10a** (0.5 mmol) in 5 ml of DCE and molecular sieves were placed in a round bottom flask with a stir bar and heated to 55 °C. Then 2 mmol of acyl Meldrum's acid **9a**–**g** were added in 4 portions every 1 h. The formation of enamide was monitored by TLC. When the spot of enaminone was no longer observed, DCE was evaporated. The final product was isolated by flash column chromatography C:M 120:1.

### Supplementary Information

Supplementary Information.

## Data Availability

The datasets presented in the current study are available from the corresponding author on reasonable request.

## References

[1] Faúndez-Parraguez M, Alarcón-Miranda C, Cho HC, Pessoa-Mahana H, Gallardo-Garrido C, Chung H, Faúndez M, Pessoa-Mahana D (2021). New pyridone-based derivatives as cannabinoid receptor type 2 agonists. Int. J. Mol. Sci..

[2] Huffman JW, Lu J, Hynd G, Wiley JL, Martin BR (2001). A pyridone analogue of traditional cannabinoids. A new class of selective ligands for the CB2 receptor. Bioorg. Med. Chem..

[3] Aytemir MD, Uzbay T, Erol DD (1999). New 4(1H)-pyridinone derivatives as analgesic agents. Arzneimittelforschung.

[4] Öztürk G, Erol DD, Aytemir MD, Uzbay T (2002). New analgesic and antiinflammatory agents 4(1H)-pyridinone derivatives. Eur. J. Med. Chem..

[5] Li R, Xue L, Zhu T, Jiang Q, Cui X, Yan Z, McGee D, Wang J, Gantla VR, Pickens JC, McGrath D, Chucholowski A, Morris SW, Webb TR (2006). Design and synthesis of 5-aryl-pyridone-carboxamides as inhibitors of anaplastic lymphoma kinase. J. Med. Chem..

[6] Lv Z, Zhang Y, Zhang M, Chen H, Sun Z, Geng D, Niu C, Li K (2013). Design and synthesis of novel 2′-hydroxy group substituted 2-pyridone derivatives as anticancer agents. Eur. J. Med. Chem..

[7] Kamauchi H, Kimura Y, Ushiwatari M, Suzuki M, Seki T, Takao K, Sugita Y (2021). Synthesis and antifungal activity of polycyclic pyridone derivatives with anti-hyphal and biofilm formation activity against *Candida*
*albicans*. Bioorg. Med. Chem. Lett..

[8] Sims GK, Sommers LE (1985). Degradation of pyridine derivatives in soil. J. Environ. Qual..

[9] Ensign JC, Rittenberg SC (1963). A crystalline pigment produced from 2-hydroxypyridine by *Arthrobacter*
*crystallopoietes* n.sp. Arch. Mikrobiol..

[10] Ryczkowska M, Maciejewska N, Olszewski M, Witkowska M, Makowiec S (2022). Design, synthesis, and biological evaluation of tetrahydroquinolinones and tetrahydroquinolines with anticancer activity. Sci. Rep..

[11] Ryczkowska M, Maciejewska N, Olszewski M, Witkowska M, Makowiec S (2022). Tetrahydroquinolinone derivatives exert antiproliferative effect on lung cancer cells through apoptosis induction. Sci. Rep..

[12] Lin S, Liu C, Zhao X, Han X, Li X, Ye Y, Li Z (2022). Recent advances of pyridinone in medicinal chemistry. Frot. Chem..

[13] Fried J, Elderfield RC (1941). Studies on lactones related to the cardiac aglycones. V. Synthesis of 5-alkyl-α-pyrones. J. Org. Chem..

[14] Konno K, Hashimoto K, Ohfune Y, Shirahama H, Matsumoto T (1988). Acromelic acids A and B. Potent neuroexcitatory amino acids isolated from *Clitocybe*
*acromelalga*. J. Am. Chem. Soc..

[15] Nagano H, Nawata Y, Hamana M (1987). Reactions of nicotinic acid 1-oxide with propionic, phenylacetic and benzoic anhydrides. Chem. Pharm. Bull..

[16] Schmidt G, Reber S, Bolli MH, Abele S (2012). Practical and scalable synthesis of S1P1 receptor agonist ACT-209905. Org. Process Res. Dev..

[17] Henecka H (1949). β-Dicarbonyl compounds. V. The condensation of acetoneoxalic ester and O-ethylacetoneoxalic ester with cyanoacetamide. Chem. Ber..

[18] Elvidge, J. A. & Zaidi, N. A. Heterocyclic syntheses with malonyl chloride. Part IX. 2-Substituted 4-chloro-6-pyrimidones from certain nitriles. *J. Chem. Soc. C*, 2188–2198 (1968).

[19] Hung NC, Bisagni E (1984). A general route to 5- and 6-substituted 4-amino-2-oxo-1,2-dihydropyridines. Synthesis.

[20] Abaszadeh M, Sheibani H, Saidi K (2009). The reaction of (chlorocarbonyl)phenyl ketene with enaminones: A novel synthesis of some 5-acyl-4-hydroxy-2- (1H)-pyridinones and 7-hydroxy-5-oxo-1,4-diazepin derivative. J. Heterocycl. Chem..

[21] Guillemont J, Benjahad A, Oumouch S, Decrane L, Palandjian P, Vernier D, Queguiner L, Andries K, de Bethune MP, Hertogs K, Grierson DS, Nguyen CH (2009). Synthesis and biological evaluation of C-5 methyl substituted 4-arylthio and 4-aryloxy-3-iodopyridin-2(1H)-one type anti-HIV agents. J. Med. Chem..

[22] Pettit GR, Fleming WC, Paull KD (1968). Synthesis of the 6- and 7-hydroxy-5,8-dioxocarbostyrils. J. Org. Chem..

[23] Fernández M, de la Cuesta E, Avendaño C (1994). Synthesis of 5-methoxy-2(1H)-quinolinone. Heterocycles.

[24] Stokes, S. *et al*. Preparation of spirocyclohexane derivatives, pharmaceutical compositions containing them and their uses as anti-apoptotic inhibitors. WO2022152705A1 (2022).

[25] Gatta F, Del Giudice MR, Pomponi M, Marta M (1992). Synthesis of 1,2,3,4-tetrahydroacridine and 5,6,7,8-tetrahydroquinoline derivatives as potential acetylcholinesterase inhibitors. Heterocycles.

[26] Huilai, Y., Jie, M. & Xuexi, S. A method of preparing of blonanserin. CN 104447551 (2015).

[27] Sakurai A, Midorikawa H (1968). The cyclization of ethyl acetoacetate and ketones by ammonium acetate. Bull. Chem. Soc. Jpn..

[28] Chen G, Zhuang Z, Li GC, Saint-Denis TG, Hsiao Y, Joe CL, Yu JQ (2017). Ligand-enabled β-C–H arylation of α-amino acids without installing exogenous directing groups. Angew. Chem. Int. Ed..

[29] Zakaszewska A, Najda-Mocarska E, Makowiec S (2017). A new approach to the stereoselective synthesis of trans-3-carbamoyl-b-lactam moieties. N. J. Chem..

[30] Zakaszewska A, Najda-Mocarska E, Makowiec S (2017). Evidence for an umpolung type of [2+2] cycloaddition of 2-carbamoyl ketenes. N. J. Chem..

[31] Janikowska K, Pawelska N, Makowiec S (2011). One step synthesis of b-lactams with retro-amide side chain. Synthesis.

[32] Szewczyk M, Punda P, Janikowska K, Makowiec S (2019). Design, synthesis, and molecular docking of new 5-HT reuptake inhibitors based on modified 1,2-dihydrocyclopenta[b]indol-3(4H)-one scaffold. J. Chem. Sci..

[33] Lipson VV, Gorobets NY (2009). One hundred years of Meldrum’s acid: Advances in the synthesis of pyridine and pyrimidine derivatives. Mol. Divers..

[34] Dumas AM, Fillion E (2010). Meldrum’s acids and 5-alkylidene Meldrum’s acids in catalytic carbon-carbon bond-forming processes. Acc. Chem. Res..

[35] Prajakta NN, Nabil AHA, Radhika SK (2015). Beckmann rearrangement for the synthesis of derivatives of β- and γ-carbolinones, dihydropyrrolopyridinone and tetrahydroisoquinolinone. ARKIVOC.

[36] Maertens F, Toppet S, Hoornaert GJ, Compernolle F (2005). Incorporation of an indole-containing diarylbutylamine pharmacophore into furo[2,3-a]carbazole ring systems. Tetrahedron.

[37] Tunbridge GA, Oram J, Caggiano L (2013). Design, synthesis and antiproliferative activity of indole analogues of indanocine. Med. Chem. Commun..

[38] Marcos A, Pedregal C, Avendaño C (1994). Synthesis of 2- and 4-oxo-1H-1-azaanthracene-9,10-diones from 2-amino-1,4-naphthoquinone. Tetrahedron.

[39] Angelov P, Velichkova S, Yanev P (2021). 4-Aminoalkyl quinolin-2-one derivatives via Knorr cyclisation of ω-amino-β-keto anilides. Molbank.

[40] Gao WT, Hou WD, Zheng MR, Tang LJ (2010). Clean and convenient one-pot synthesis of 4-hydroxycoumarin and 4-hydroxy-2-quinolinon derivatives. Synth. Commun..

[41] Park SJ, Lee JC, Lee KI (2007). A facile synthesis of 4-hydroxycoumarin and 4-hydroxy-2-quinolone derivative. Bull. Korean Chem. Soc..

[42] Chemaxon pKa calculator. https://chemaxon.com/calculators-and-predictors

[44] Khopade TM, Warghude PK, Mete TB, Bhat RG (2019). Acyl/aroyl Meldrum’s acid as an enol surrogate for the direct organocatalytic synthesis of α, β unsaturated ketones. Tetrahedron Lett..

[45] Emtenäs H, Alderin L, Almqvist F (2001). An enantioselective ketene-imine cycloaddition method for synthesis of substituted ring-fused 2-pyridinones. J. Org. Chem..

[43] Pita B, Masaguer CF, Raviña E (2002). New synthetic approaches to CNS drugs. A straightforward, efficient synthesis of tetrahydroindol-4-ones and tetrahydroquinolin-5-ones via palladium-catalyzed oxidation of hydroxyenaminones. Tetrahedron Lett..

[46] Chandran R, Pise A, Shah SK, Rahul D, Suman V, Tiwari KN (2020). Copper-catalyzed thiolation of terminal alkynes employing thiocyanate as the sulfur source leading to enaminone-based alkynyl sulfides under ambient conditions. Org. Lett..

