# Supplementary material for: Meldrum’s acid assisted formation of tetrahydroquinolin-2-one derivatives a short synthetic pathway to the biologically useful scaffold
Source: Sci Rep. 2024 Jan 4;14:487. doi: 10.1038/s41598-023-50535-0 (PMC10766601; doi:10.1038/s41598-023-50535-0)
Supplement: Supplementary file 1 — Supplementary Information. [file 41598_2023_50535_MOESM1_ESM.pdf]

SUPPLEMENTARY INFORMATION

Synthetic return of the Meldrum's acid. 2,2-Dimethyl-1,3-dioxane-4,6-dione assisted formation of tetrahydroquinolin-2-one derivatives. Short synthetic pathway to the biologically useful scaffold.

Małgorzata Ryczkowska[1], Alicja Trocka[1], Anna Hromova[1] and Sławomir Makowiec[1]*

[1] Department of Organic Chemistry, Faculty of Chemistry, Gdansk University of Technology, Narutowicza 11/12, 80-233, Gdansk, Poland
*mak@pg.edu.pl

TABLE OF CONTENTS

EXPERIMENTAL DATA 3
GENERAL 3
A. Procedure for 1,6,7,8-tetrahydroquinoline-2,5-dione derivatives 3
1. 7,7-dimethyl-4-phenyl-6,8-dihydro-1H-quinoline-2,5-dione (12aa) 3
2. 7,7-dimethyl-4-[3-(trifluoromethyl)phenyl]-6,8-dihydro-1H-quinoline-2,5-dione (12ba) 5
3. 9-hydroxy-2,6-bis(4-methoxyphenyl)-11,11-dimethyl-10,12-dihydro-[1,3]oxazino[2,3-j]quinoline-4,8-dione (13ca) 6
4. 4,7,7-trimethyl-6,8-dihydro-1H-quinoline-2,5-dione (12da) 12
5. 4-ethyl-7,7-dimethyl-6,8-dihydro-1H-quinoline-2,5-dione (12ea) 13
6. 4-phenyl-1,6,7,8-tetrahydroquinoline-2,5-dione (12ab) 15
7. 7-methyl-4-phenyl-1,6,7,8-tetrahydroquinoline-2,5-dione (12ac) 16
8. 4,7-diphenyl-1,6,7,8-tetrahydroquinoline-2,5-dione (12ad) 18
9. 2,6-bis(2-furyl)-9-hydroxy-11,11-dimethyl-10,12-dihydro-[1,3]oxazino[2,3-j]quinoline-4,8-dione (13ga) 19
B. Procedure for 5-(5,5-dimethyl-3-oxo-cyclohexen-1-yl)-3-oxo-propanamide derivatives 21
1. 5-(5,5-dimethyl-3-oxo-cyclohexen-1-yl)-3-oxo-3-phenyl-propanamide (11aa) 21
2. 5-(5,5-dimethyl-3-oxo-cyclohexen-1-yl)-3-(4-methoxyphenyl)-3-oxo-propanamide (11ca) 23
3. 5-(5,5-dimethyl-3-oxo-cyclohexen-1-yl)-3-(2-furyl)-3-oxo-propanamide (11ga) 24
REFERANCES 26

EXPERIMENTAL DATA

GENERAL

Commercially available reagents were purchased from Sigma-Aldrich or Acros and used without further purification. Acyl Meldrum's acids **9a-f** and enaminones **10a-d** were prepared according to literature procedures; **9a**, **9b**, **9c**, **9f** [1], **9d**, **9e** [2], **10a-d** [3].

Analytical thin-layer chromatography was performed on aluminum sheets of UV-254 Merck silica gel, and flash chromatography using SilicaFlash P60 silica gel (40–63 µm). ^{1}H and ^{13}C NMR spectra were recorded with Bruker Avance III HD 400 MHz or Varian Gemini 500 MHz and NMR chemical shifts were reported in δ (ppm) using residual solvent peaks as standards, with the coupling constant J measured in Hz. High-resolution mass spectra were recorded with an Agilent 6540 Q-TOF system. High-resolution (HRMS) was recorded on Agilent 6540 Q-TOF.

A. Procedure for 1,6,7,8-tetrahydroquinoline-2,5-dione derivatives

A solution of enaminone (0.5 mmol) in 5 ml of DCE and molecular sieves were placed in a round-bottom flask with a stir bar and heated to 55°C. Then 2 mmol of acyl Meldrum's acid were added in 4 portions every 1 hour. The formation of enamide was monitored by TLC. When the spot of enaminone was no longer observed, 0.8 g of PPA was added. The reaction mixture was then heated to reflux (84 °C), left for 6h and after that, DCE was evaporated. The residue was then suspended in water, cooled in the ice bath, and neutralized with NaOH. Next, the resulting suspension was subjected to extraction with AcOEt and DCM. Organic layers were washed with brine and dried with anhydrous $MgSO_4$. The final product was isolated by flash column chromatography (C:M 200:1 and if needed A:H 2:1). All of the title compounds were obtained as white amorphous powders.

1. 7,7-dimethyl-4-phenyl-6,8-dihydro-1H-quinoline-2,5-dione (12aa)

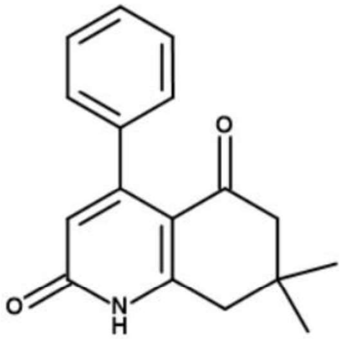

Yield: 34%,

^{1}H NMR ($CDCl_3$, 400 MHz): δ= 13.21 (s, NH), 7.39 – 7.38 (m, 3 H), 7.22 – 7.20 (m, 2 H), 6.36 (s, 1 H), 2.92 (s, 2 H), 2.41 (s, 2 H), 1.16 (s, 6 H)

^{13}C NMR ($CDCl_3$, 125 MHz): δ= 193.46, 164.72, 156.55, 155.57, 139.36, 128.29, 127.99, 127.40, 113.87, 119.40, 52.84, 41.86, 32.90, 28.21

HRMS (ESI+): *m*/*z*[M + H]+ calcd for $C_{17}H_{18}NO_2$: 268.1337; found: 268.1333

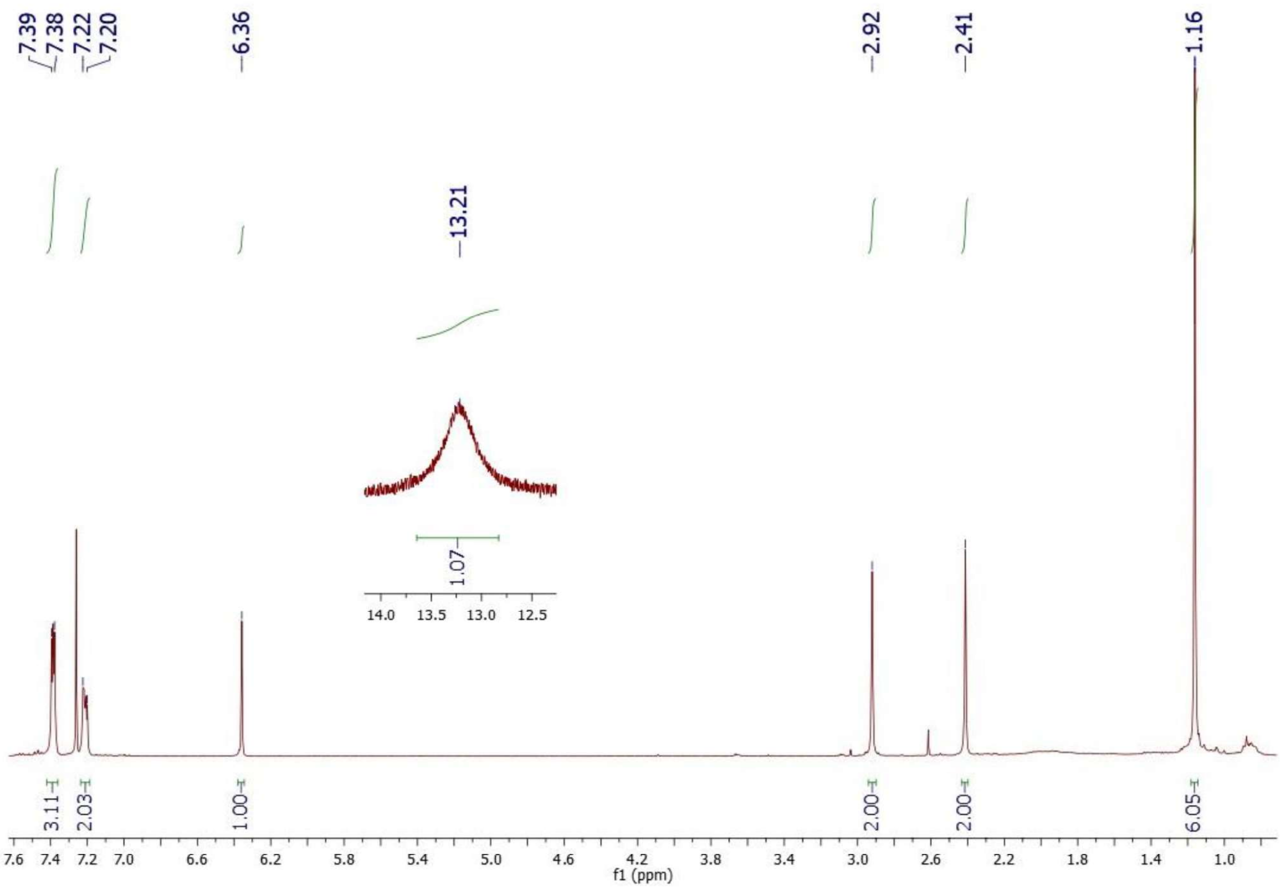

Figure S1. ^{1}H NMR of 7,7-dimethyl-4-phenyl-6,8-dihydro-1H-quinoline-2,5-dione

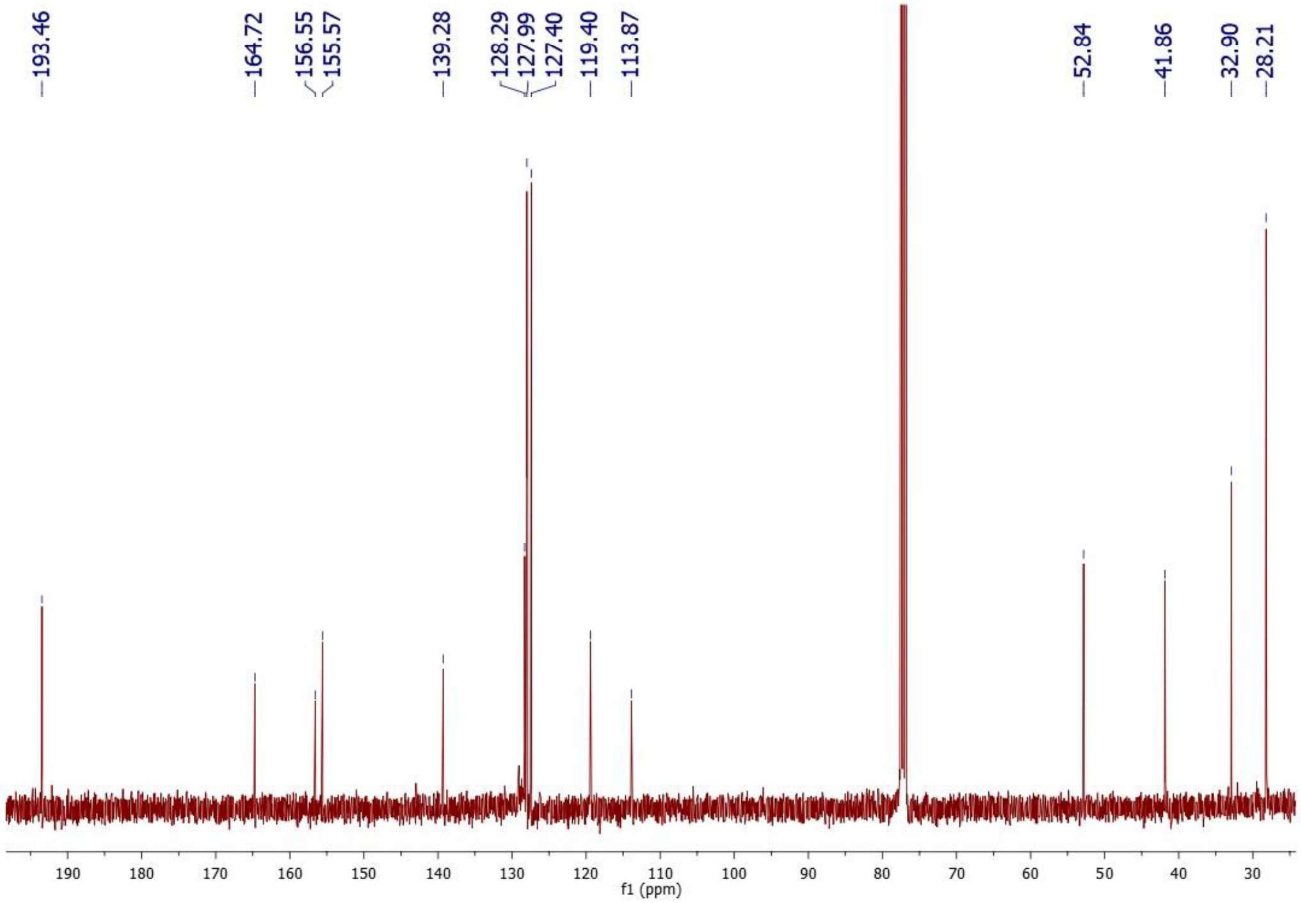

Figure S2. ^{13}C NMR of 7,7-dimethyl-4-phenyl-6,8-dihydro-1H-quinoline-2,5-dione

2. 7,7-dimethyl-4-[3-(trifluoromethyl)phenyl]-6,8-dihydro-1H-quinoline-2,5-dione (12ba)

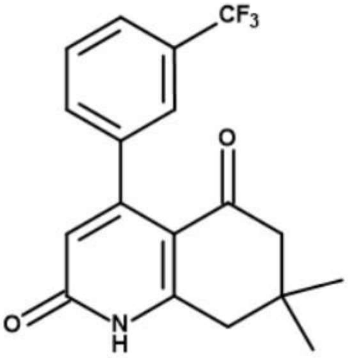

Yield: 29%,

^{1}H NMR ($CDCl_3$, 400 MHz): δ= 13.34 (s, NH), 7.63 (d, J = 8 Hz, 1 H), 7.38 (d, J = 8 Hz, 1 H), 7.51 – 7.47 (m, 2 H), 6.31 (s, 1 H), 2.91 (s, 2 H), 2.40 (s, 2 H), 1.15 (s, 6 H)

^{13}C NMR ($CDCl_3$, 100 MHz): δ= 193.34, 164.81, 156.10, 154.37, 140.20, 130.94, 130.36 (q, J^2 = 32 Hz), 128.22, 124.13 (q, J^1 = 270 Hz), 124.88 (q, J^3 = 3.7 Hz), 124.24 (q, J^3 = 3.7 Hz), 120.09, 112.79, 52.64, 41.79, 32.82, 28.16

HRMS (ESI+): m/z

[M + H]+ calcd for $C_{18}H_{17}F_3NO_2$: 336.1211; found: 336.1207

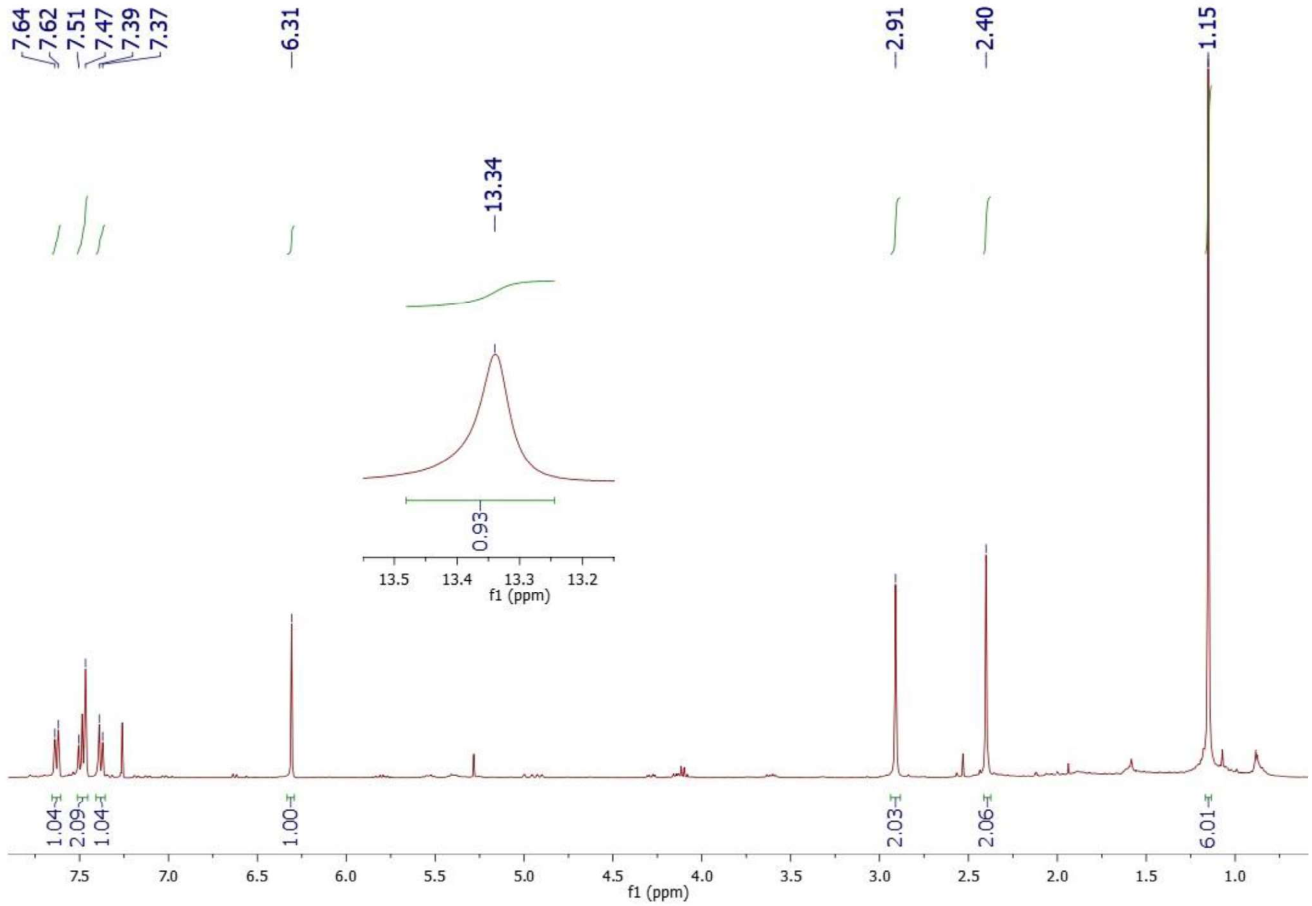

Figure S3. ^{1}H NMR of 7,7-dimethyl-4-[3-(trifluoromethyl)phenyl]-6,8-dihydro-1H-quinoline-2,5-dione

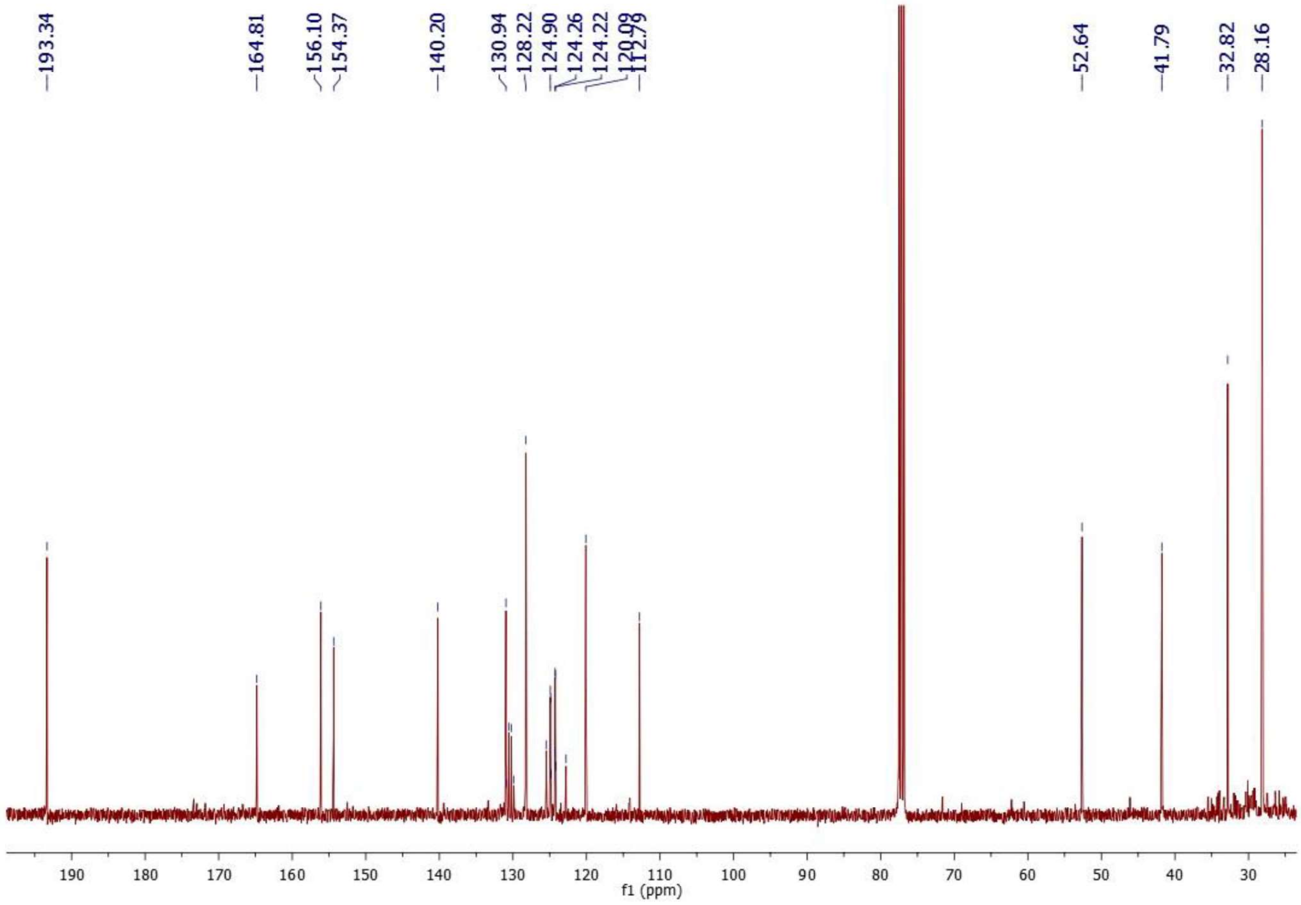

Figure S4. ^{13}C NMR of 7,7-dimethyl-4-[3-(trifluoromethyl)phenyl]-6,8-dihydro-1H-quinoline-2,5-dione

3. 9-hydroxy-2,6-bis(4-methoxyphenyl)-11,11-dimethyl-10,12-dihydro-[1,3]oxazino[2,3-j]quinoline-4,8-dione (13ca)

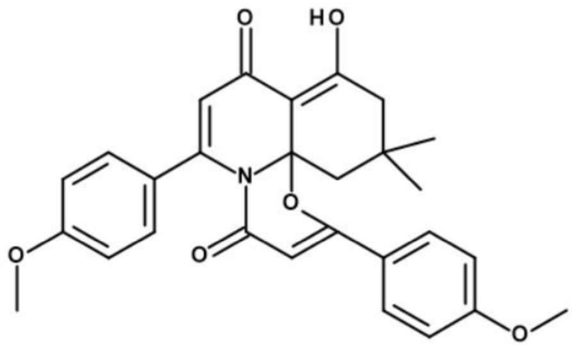

Yield: 24%,

^{1}H NMR ($CDCl_3$, 500 MHz): δ= 11.17 (s, OH), 7.74 (d, *J* = 11 Hz, 2 H), 7.61 (d, *J* = 11 Hz, 2 H), 7.00 (d, *J* = 11 Hz, 2 H), 6.98 (d, *J* = 11 Hz, 2 H), 6.93 (s, 1 H), 6.79 (s, 1 H), 3.88 (s, 3H), 3.87 (s, 3 H), 2.43 (s, 2 H), 2.22 (s, 2 H), 1, 07 (s, 6 H)

^{13}C NMR ($CDCl_3$, 125 MHz): δ= 200.30, 179.24, 170.59, 163.96, 163.19, 162.60, 161.92, 153.36, 131.10, 128.16, 124.39, 121.96, 116.45, 114.90, 114.11, 112.03, 108.84, 55.71, 55.60, 50.73, 42.86, 32.87, 28.37

HRMS (ESI+): *m/z*

[M + H]+ calcd for $C_{28}H_{28}NO_6$: 474.1917; found: 474.1916

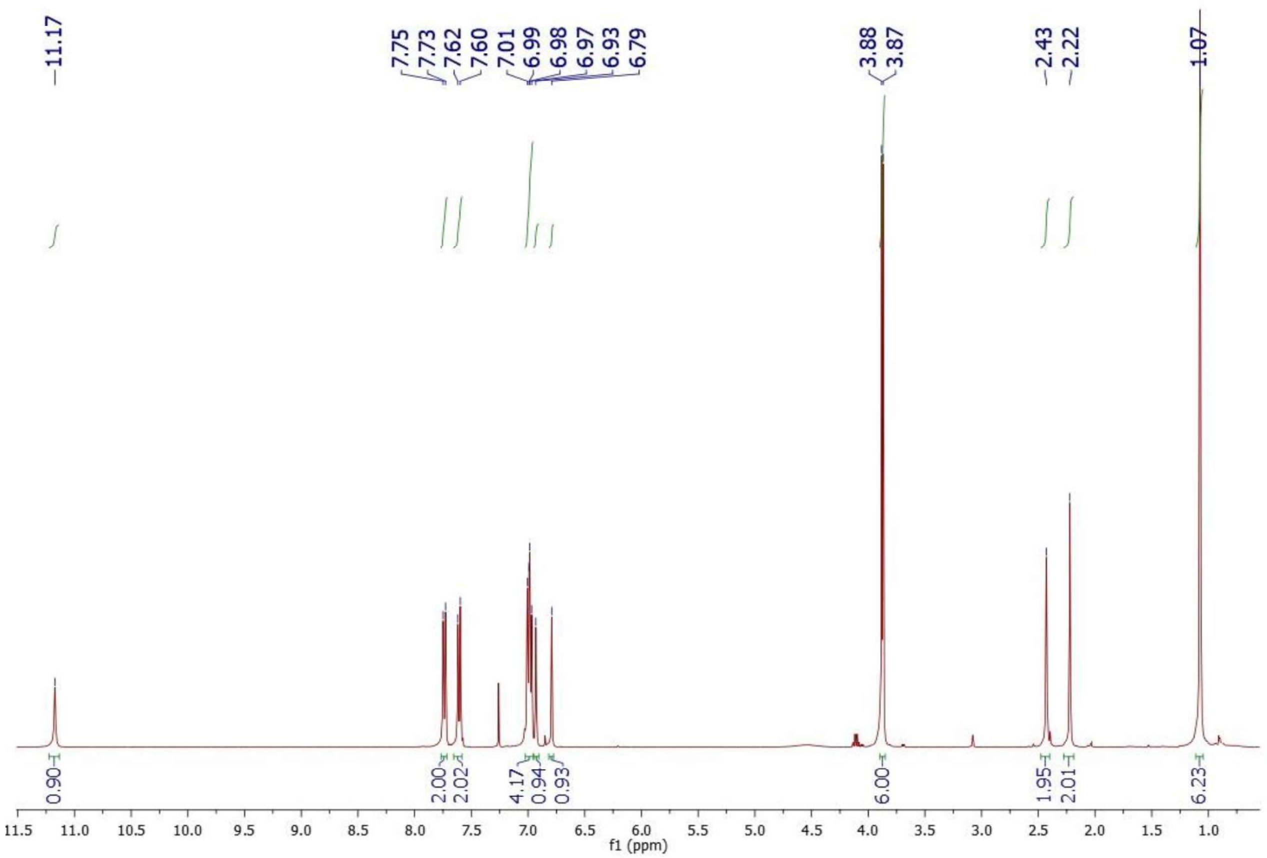

Figure S5. ^{1}H **NMR of 9-hydroxy-2,6-bis(4-methoxyphenyl)-11,11-dimethyl-10,12-dihydro-[1,3]oxazino[2,3-j]quinoline-4,8-dione**

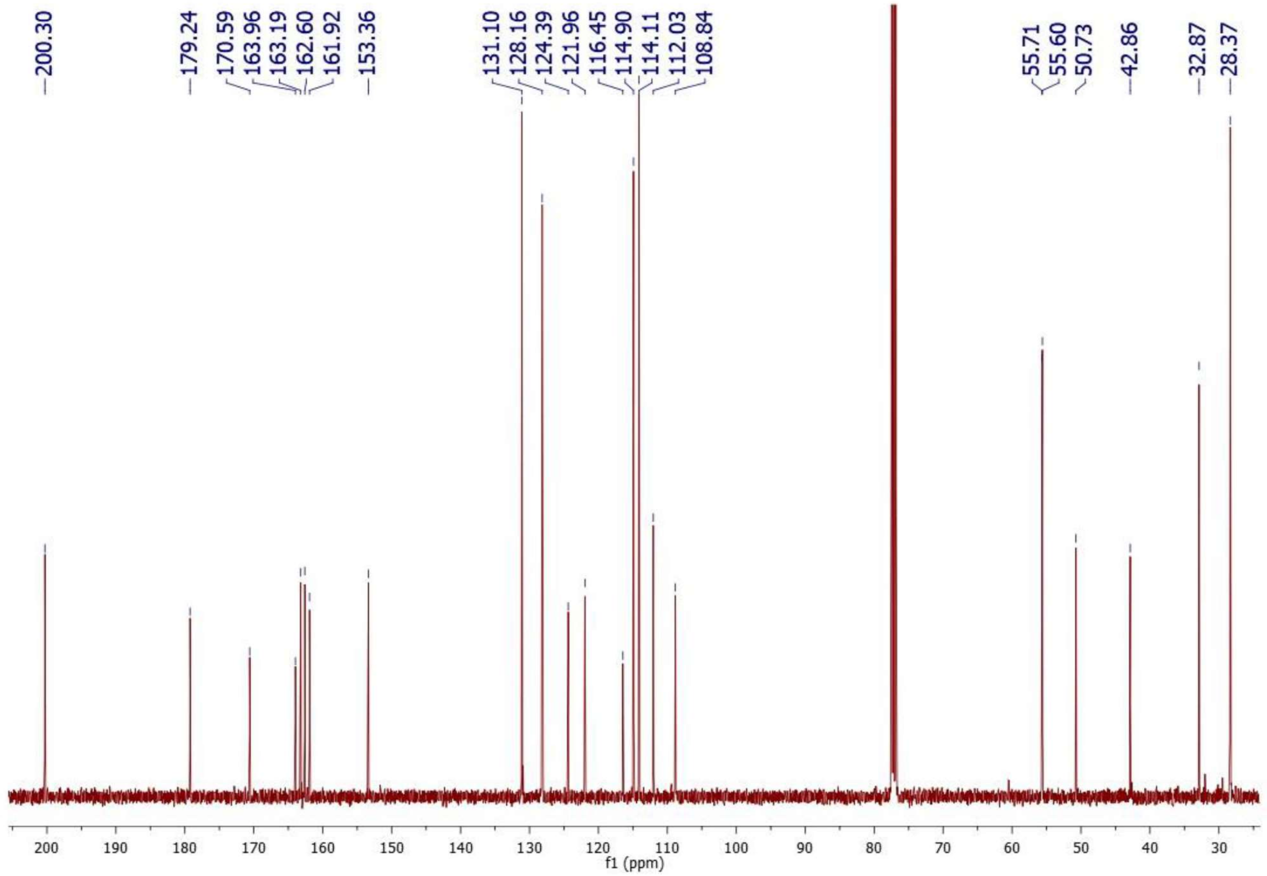

Figure S6. ^{13}C **NMR of 9-hydroxy-2,6-bis(4-methoxyphenyl)-11,11-dimethyl-10,12-dihydro-[1,3]oxazino[2,3-j]quinoline-4,8-dione**

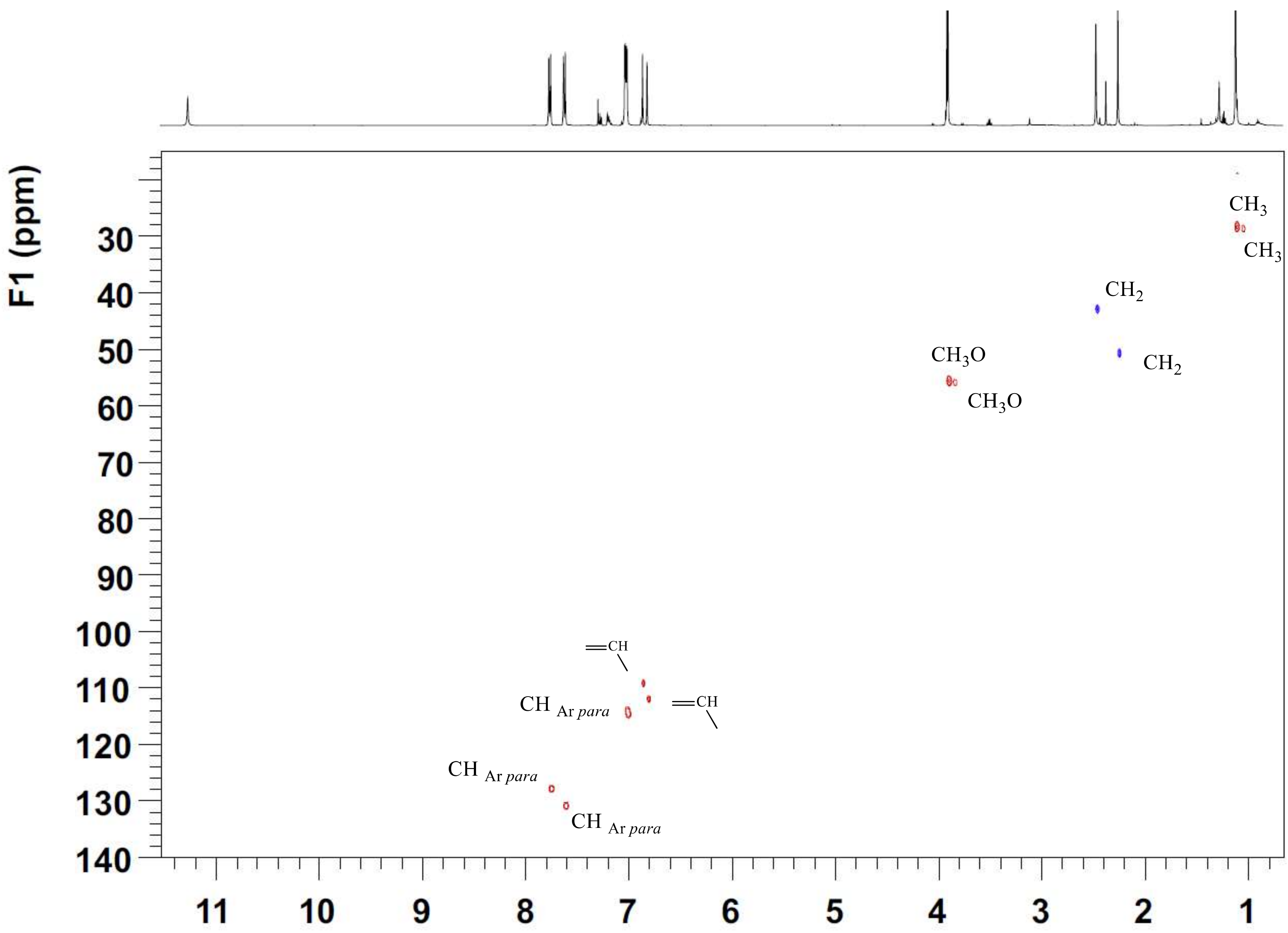

Figure S7. HSQC NMR of 9-hydroxy-2,6-bis(4-methoxyphenyl)-11,11-dimethyl-10,12-dihydro-[1,3]oxazino[2,3-j]quinoline-4,8-dione

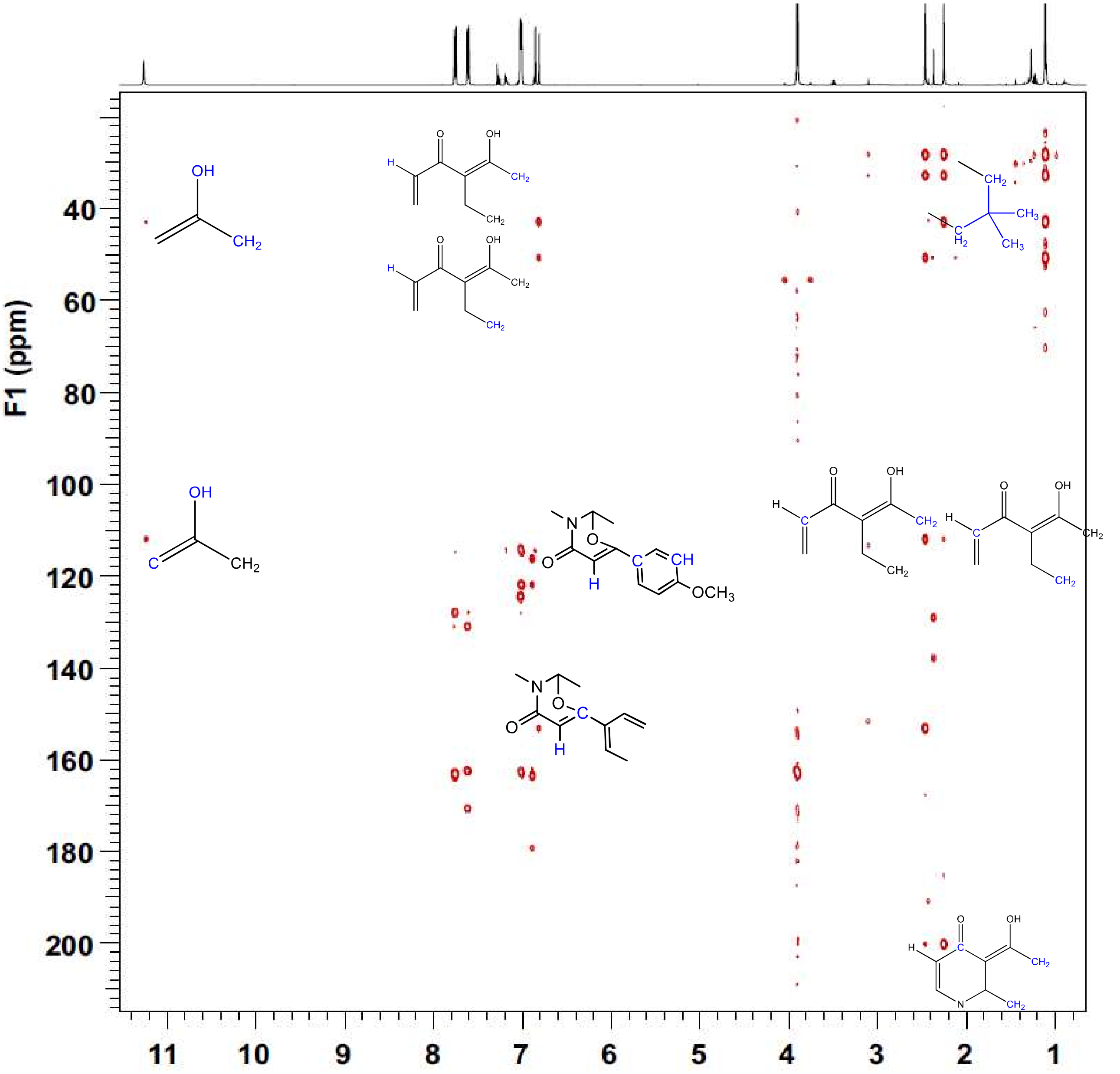

Figure S8. HMBC NMR of 9-hydroxy-2,6-bis(4-methoxyphenyl)-11,11-dimethyl-10,12-dihydro-[1,3]oxazino[2,3-j]quinoline-4,8-dione

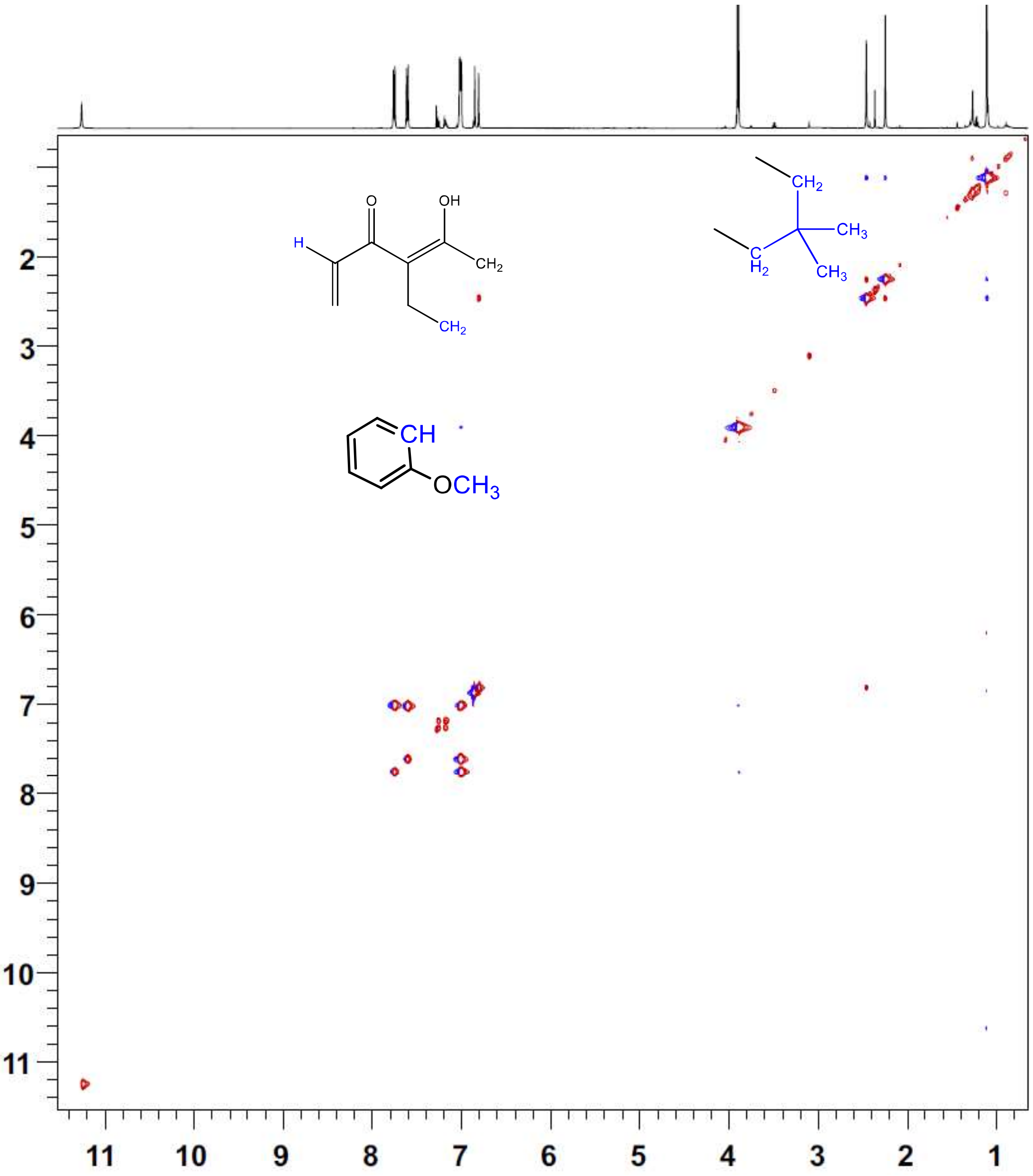

Figure S9. TOCSY NMR of 9-hydroxy-2,6-bis(4-methoxyphenyl)-11,11-dimethyl-10,12-dihydro-[1,3]oxazino[2,3-j]quinoline-4,8-dione

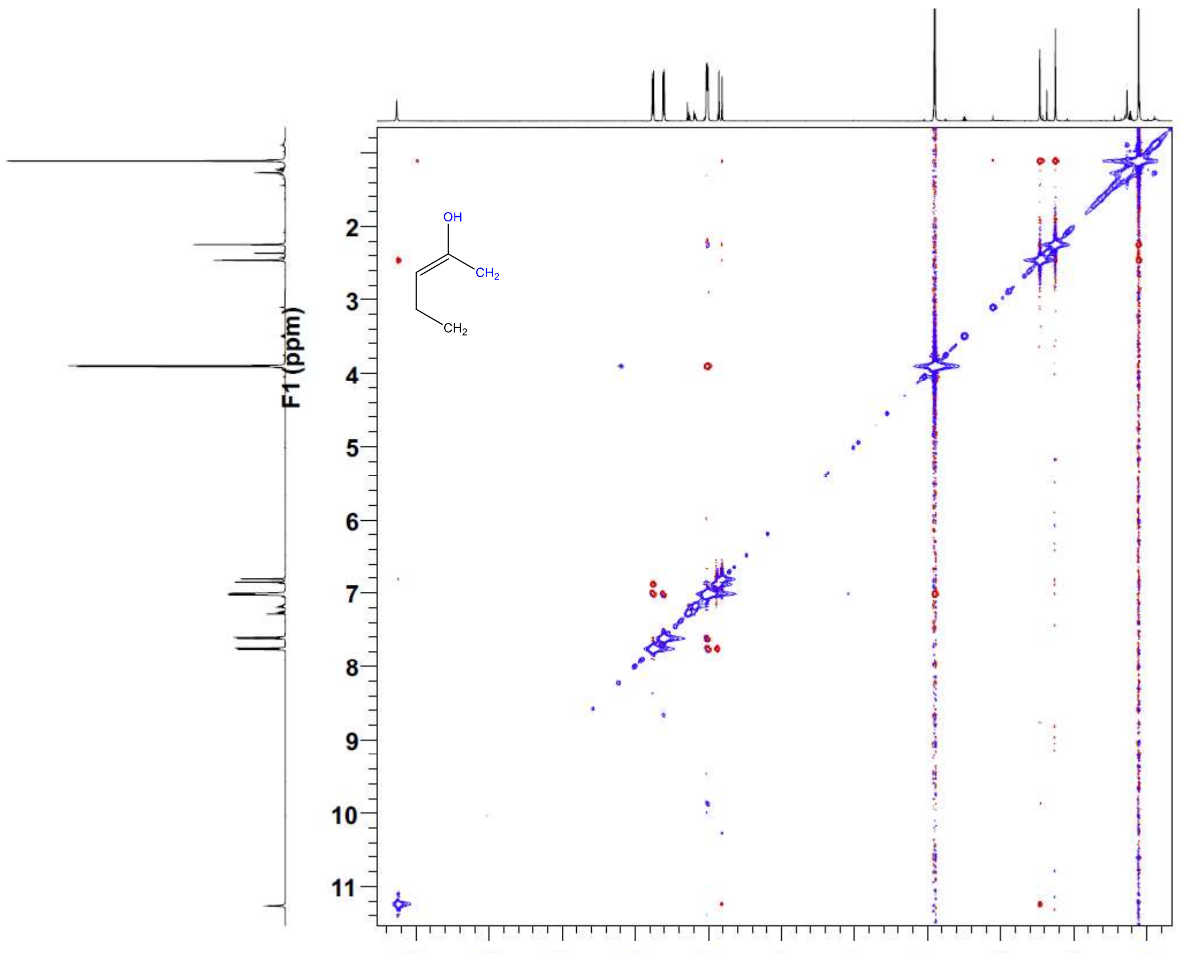

Figure S10. ROESY NMR of 9-hydroxy-2,6-bis(4-methoxyphenyl)-11,11-dimethyl-10,12-dihydro-[1,3]oxazino[2,3-j]quinoline-4,8-dione

4. 4,7,7-trimethyl-6,8-dihydro-1H-quinoline-2,5-dione (12da)

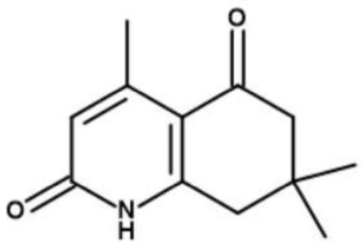

Yield: 33%,

^{1}H NMR (CDCl$_3$, 500 MHz): δ= 13.28 (s, NH), 6.26 (s, 1 H), 2.83 (s, 2 H), 2.55 (s, 3 H), 2.41 (s, 2 H), 1.11 (s, 3 H)

^{13}C NMR (CDCl$_3$, 125 MHz): δ= 195.60, 165.02, 155.46, 155.26, 118.44, 114.16, 53.13, 41.70, 32.50, 28.10, 23.43,

HRMS (ESI+): *m/z*

[M + H]+ calcd for $C_{12}H_{16}NO_2$: 206.1181; found: 206.1175

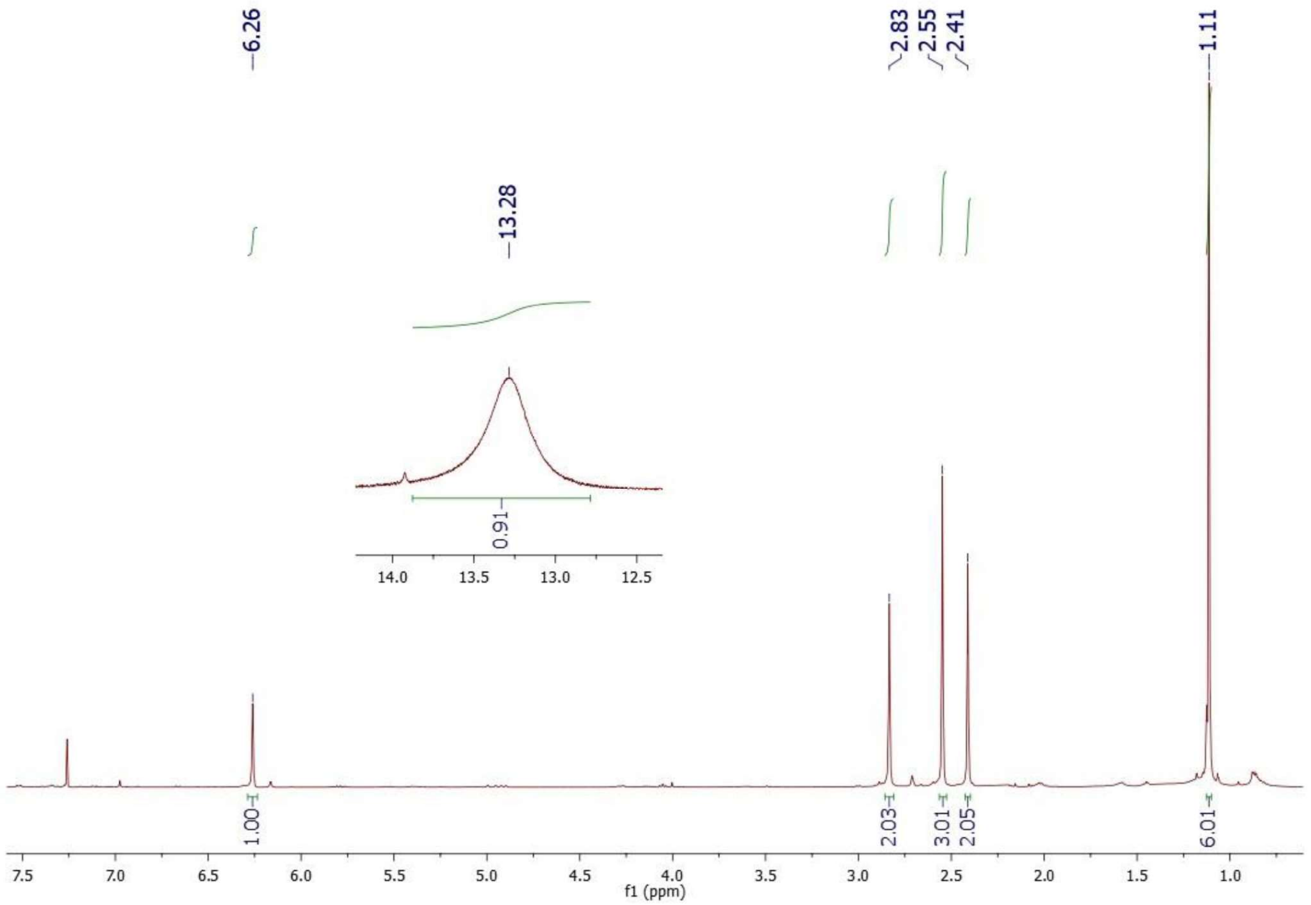

Figure S11. ^{1}H NMR of 4,7,7-trimethyl-6,8-dihydro-1H-quinoline-2,5-dione

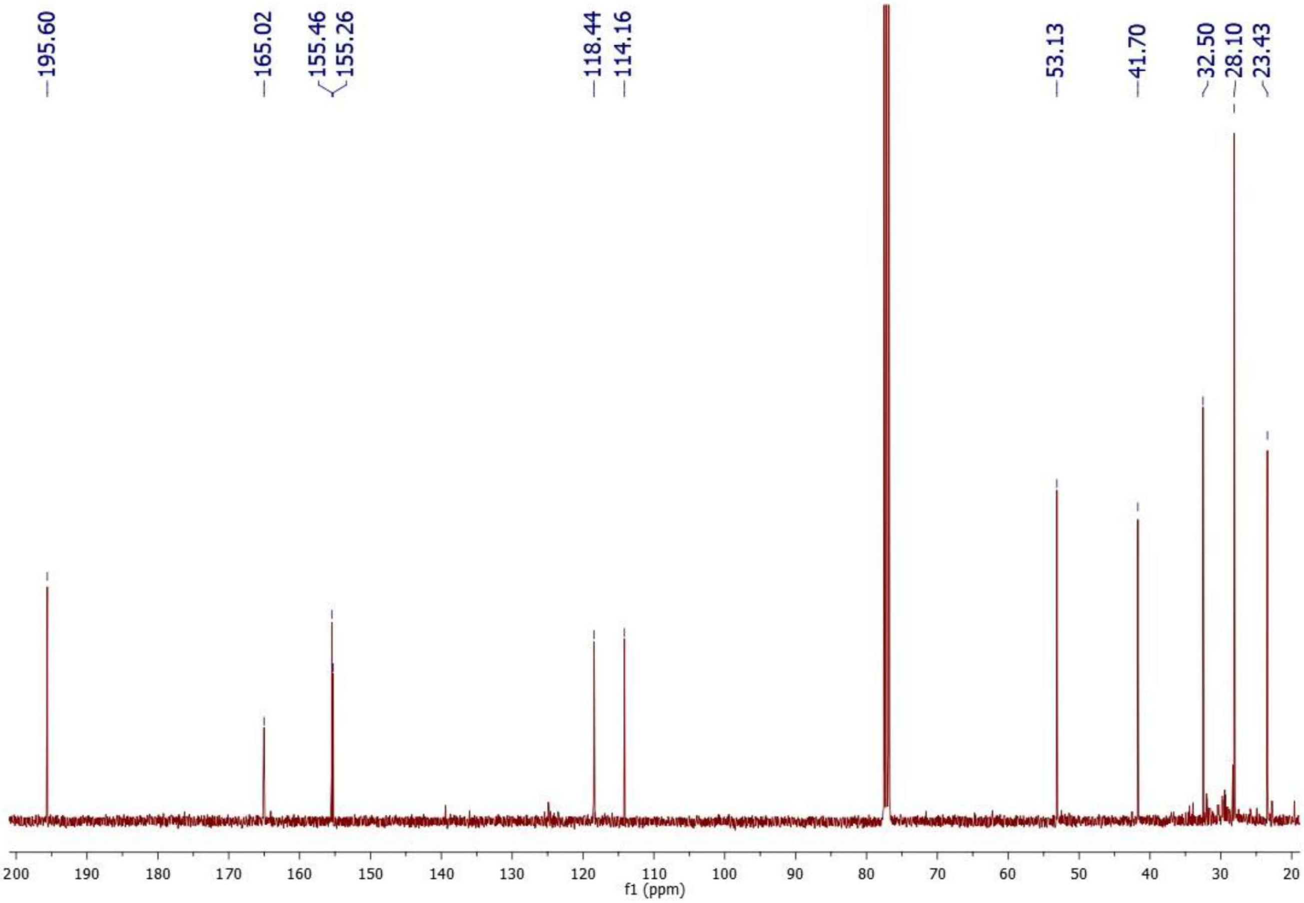

Figure S12. **^{13}C NMR of 4,7,7-trimethyl-6,8-dihydro-1H-quinoline-2,5-dione**

5. 4-ethyl-7,7-dimethyl-6,8-dihydro-1H-quinoline-2,5-dione (12ea)

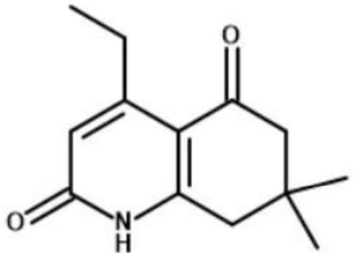

Yield: 31%,

^{1}H NMR ($CDCl_3$, 500 MHz): δ= 13.38 (s, NH), 6.36 (s, 1 H), 3.02 (q, *J* = 9.0 Hz, 2 H), 2.87 (s, 2 H), 2.42 (s, 2 H), 1.17 (t, *J* = 9 Hz, 3 H), 1.11 (s, 6 H)

^{13}C NMR ($CDCl_3$, 125 MHz): δ= 195.32, 165.15, 161.39, 155.72, 116.26, 114.04, 53.39, 41.88, 32.50, 28.32, 28.06, 13.78

HRMS (ESI+): *m*/*z*

[M + H]+ calcd for $C_{13}H_{18}NO_2$: 220.1337; found: 220.1332

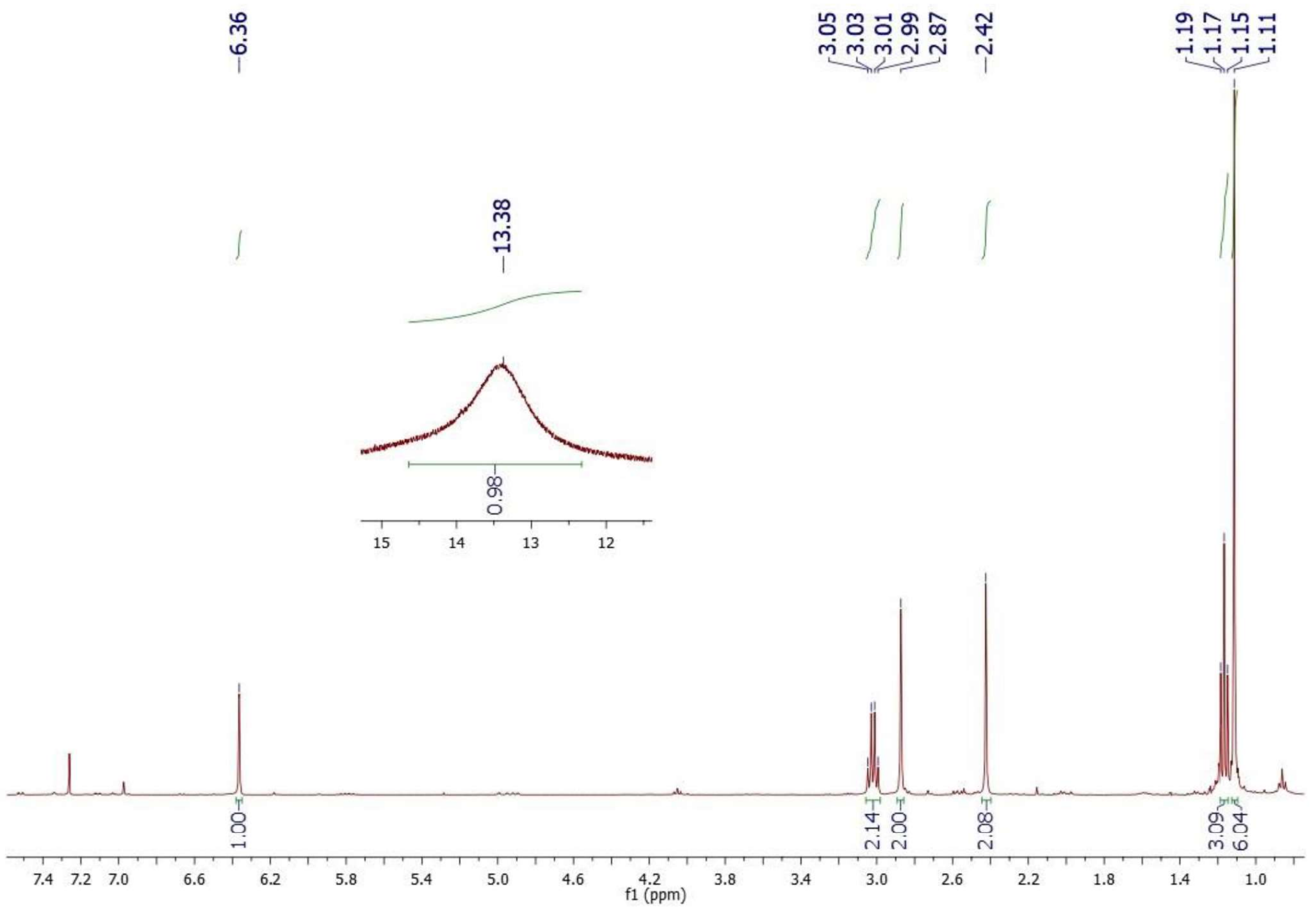

Figure S13. ^{1}H NMR of 4-ethyl-7,7-dimethyl-6,8-dihydro-1H-quinoline-2,5-dione

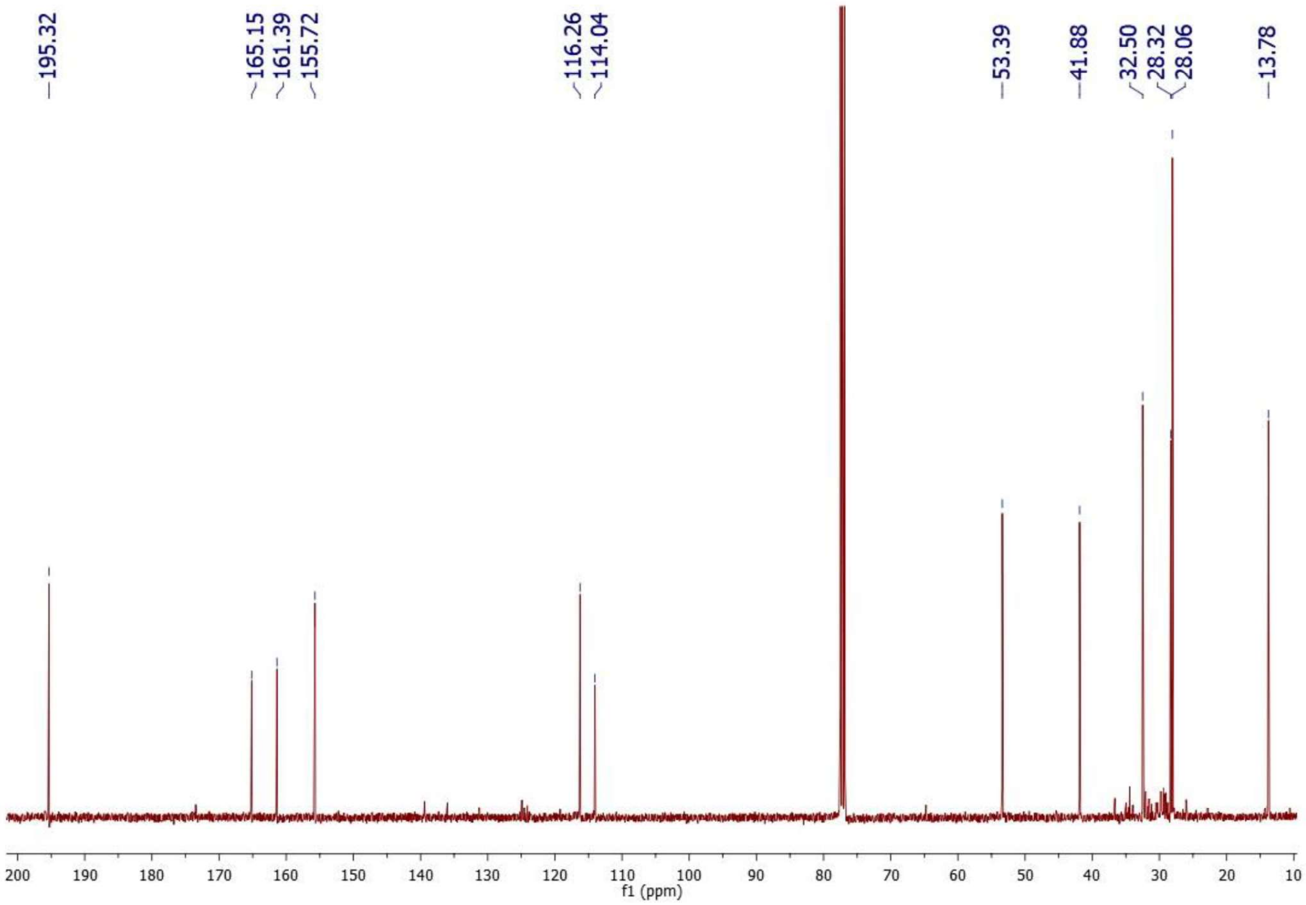

Figure S14. ^{13}C NMR of 4-ethyl-7,7-dimethyl-6,8-dihydro-1H-quinoline-2,5-dione

6. 4-phenyl-1,6,7,8-tetrahydroquinoline-2,5-dione (12ab)

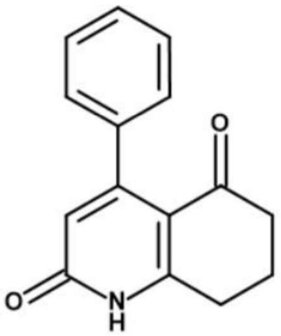

Yield: 37%,

^{1}H NMR ($CDCl_3$, 500 MHz): δ= 13.37 (s, NH), 7.40 – 7.38 (m, 3 H), 7.22 – 7.20 (m, 2 H), 6.37 (s, 1 H), 3.05 (t, *J* = 6 Hz, 2 H), 2.55 (t, *J* = 6 Hz, 2 H), 2.17 (qu, *J* = 6 Hz, 2 H)

^{13}C NMR ($CDCl_3$, 125 MHz): δ= 193.31, 163.91, 157.71, 157.18, 139.10, 128.49, 128.05, 127.32, 118.86, 115.72, 38.89, 28.43, 20.91

HRMS (ESI+): *m/z*

[M + H]+ calcd for $C_{15}H_{14}NO_2$: 240.1025; found: 240.1019

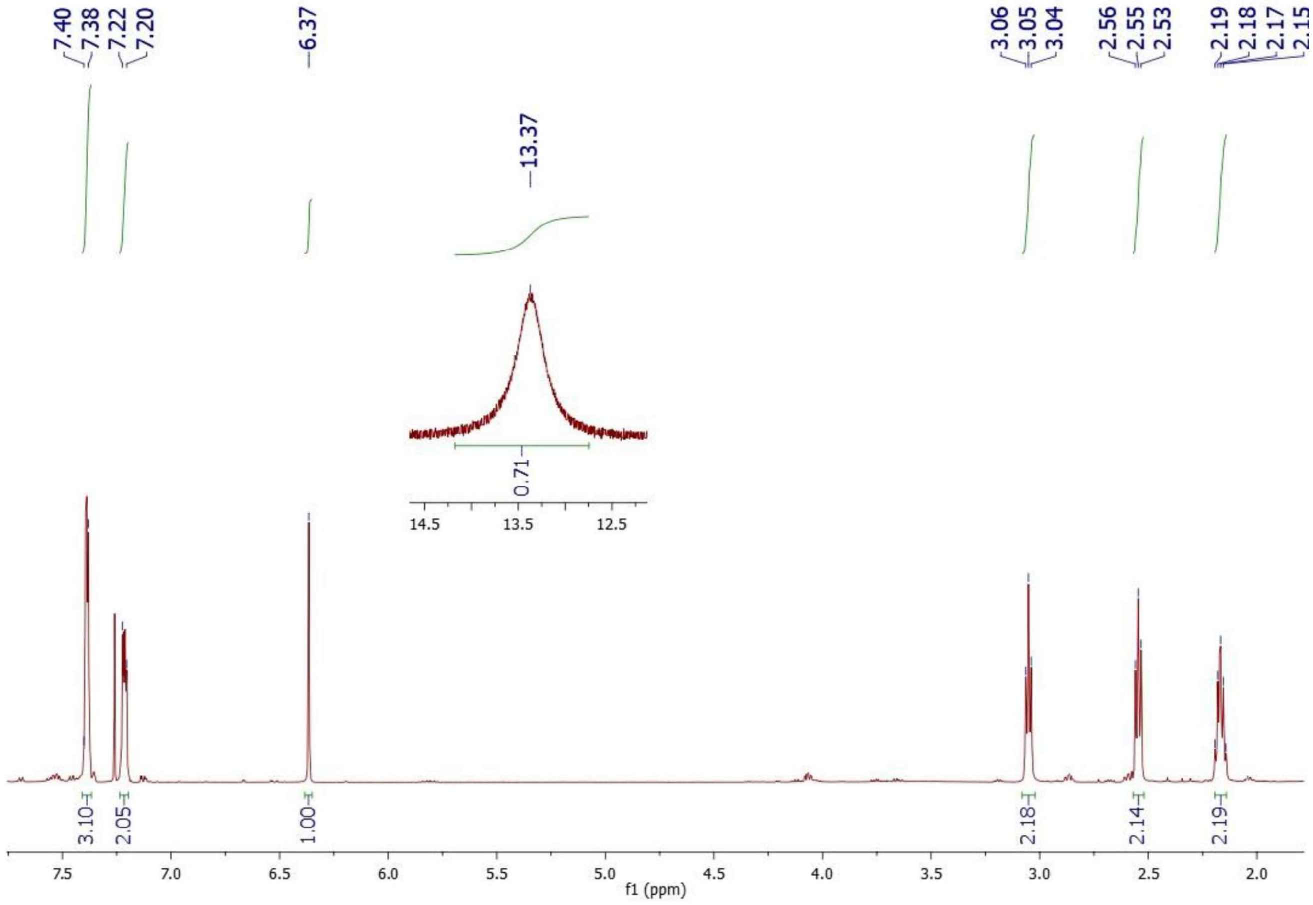

Figure S15. ^{1}H NMR of 4-phenyl-1,6,7,8-tetrahydroquinoline-2,5-dione

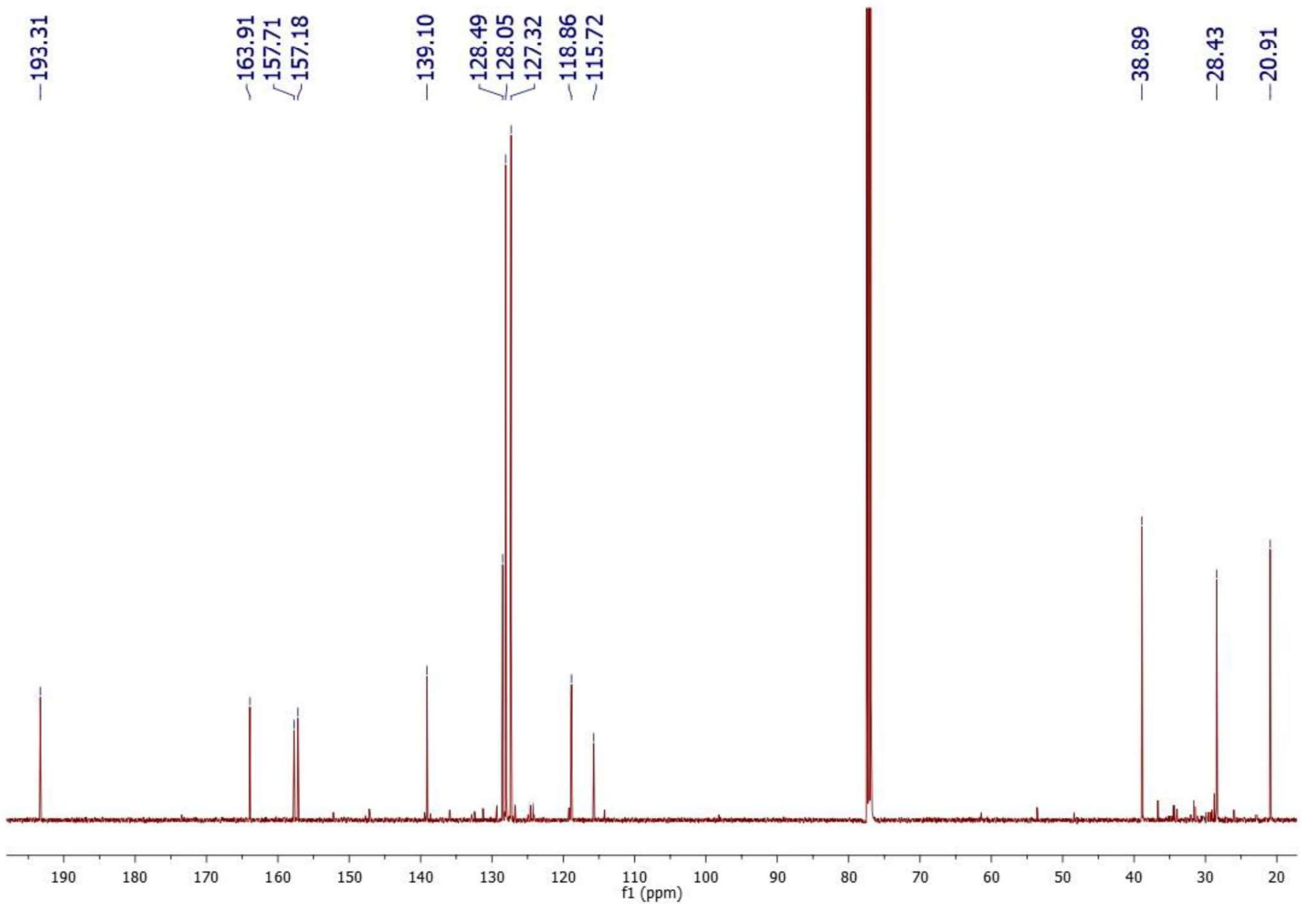

Figure S16. ^{13}C NMR of 4-phenyl-1,6,7,8-tetrahydroquinoline-2,5-dione

7. 7-methyl-4-phenyl-1,6,7,8-tetrahydroquinoline-2,5-dione (12ac)

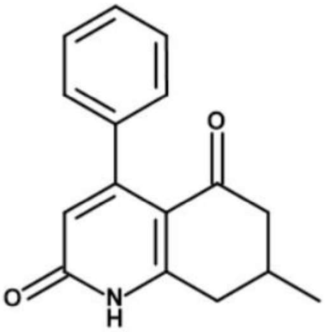

Yield: 20%,

^{1}H NMR ($CDCl_3$, 500 MHz): δ= 13.40 (s, NH), 7.40 – 7.38 (m, 3 H), 7.22 – 7.20 (m, 2 H), 6.39 (s, 1 H), 3.12 (dd, J^2 = 17 Hz, J^3 = 3.5 Hz, 1 H), 2.75 (dd, J^2 = 17 Hz, J^3 = 11 Hz), 2.60 (ddd, J^2 = 17 Hz, J^3 = 3.5 Hz, J^4 = 1 Hz, 1 H), 2.46 – 2.39 (m, 1 H), 2.25 (dd, J^2 = 17 Hz, J^3 = 11 Hz, 1 H), 1.17 (d, J = 6.5 Hz, 3 H)

^{13}C NMR ($CDCl_3$, 125 MHz): δ= 193.34, 156.50, 128.60, 128.58, 128.08, 127.36, 124.91, 47.15, 36.16, 28.66, 20.98 (expected 14, counted 11 carbon atoms)

HRMS (ESI+): *m/z*

[M + H]+ calcd for $C_{16}H_{16}NO_2$: 254.1181; found: 254.1176

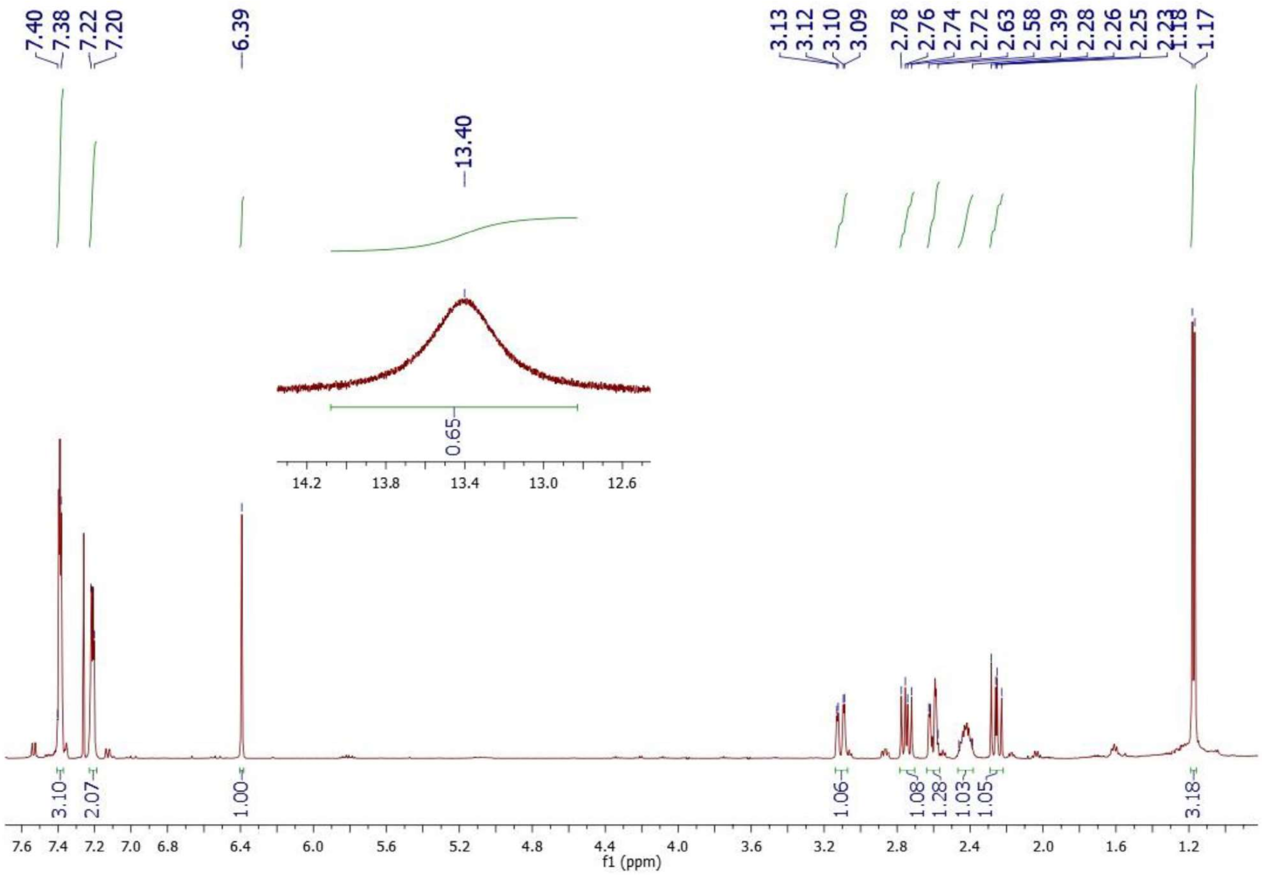

Figure S17. ^{1}H NMR of 7-methyl-4-phenyl-1,6,7,8-tetrahydroquinoline-2,5-dione

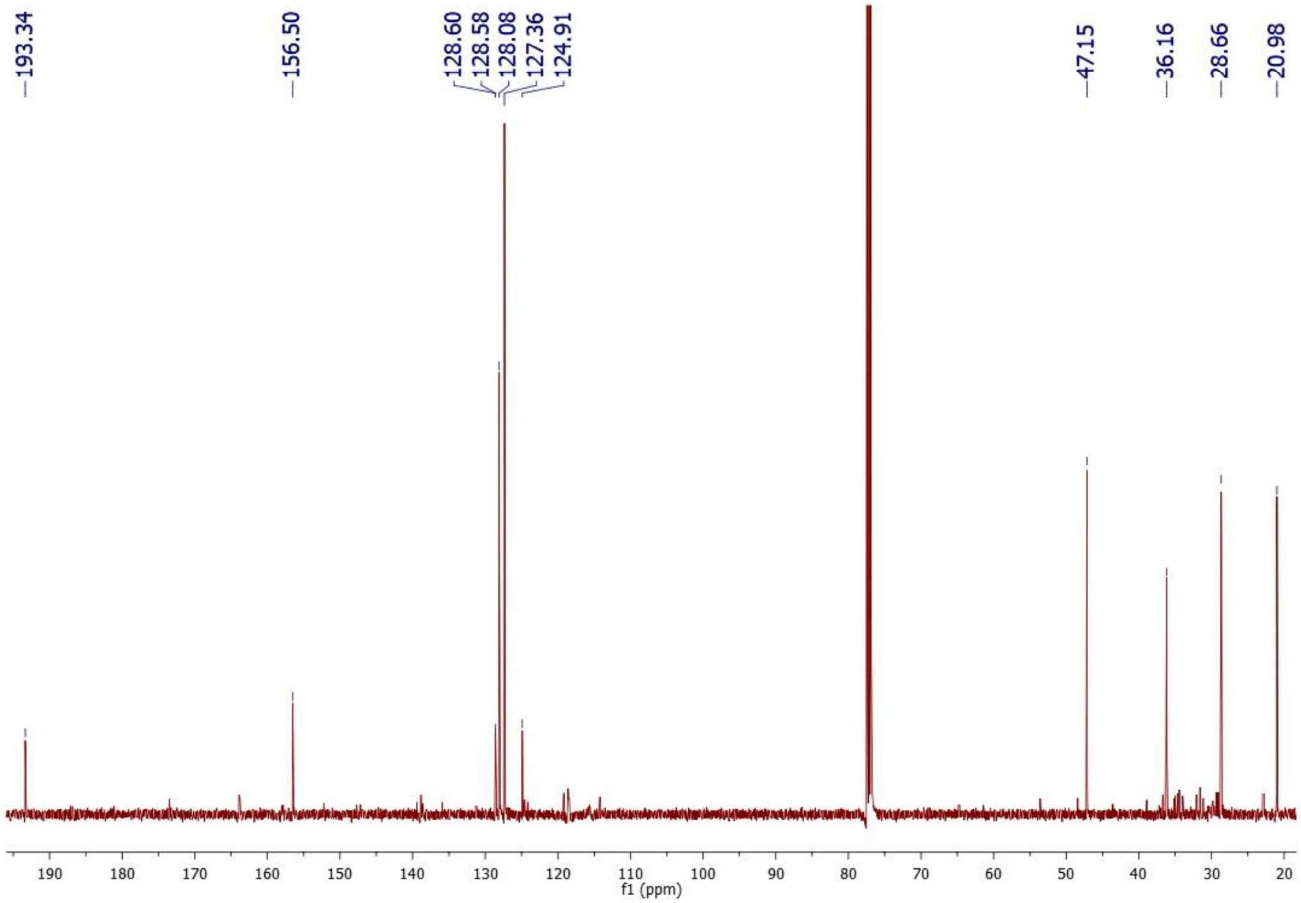

Figure S18. ^{13}C NMR of . 7- methyl-4-phenyl-1,6,7,8-tetrahydroquinoline-2,5-dione

8. 4,7-diphenyl-1,6,7,8-tetrahydroquinoline-2,5-dione (12ad)

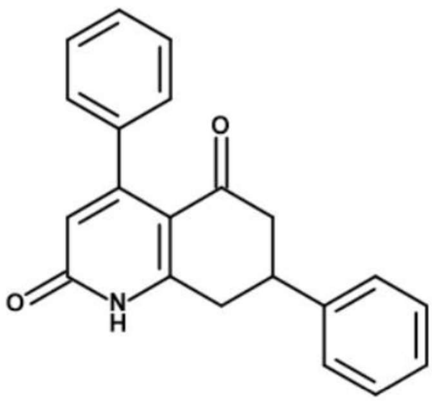

Yield: 22%,

^{1}H NMR ($CDCl_3$, 500 MHz): δ= 13.42 (s, NH), 7.39 – 7.36 (m, 5 H), 7.31 – 7.28 (m, 3 H), 7.24 – 7.21 (m, 2 H), 6.34 (s, 1 H), 3.57 – 3.51 (m, 1 H), 3.31 – 3.20 (m, 2 H), 2.83 – 2.74 (m, 2 H)

^{13}C NMR ($CDCl_3$, 125 MHz): δ= 192.67, 164.53, 156.50, 156.26, 141.59, 139.26, 129.07, 128.29, 127.96, 127.57, 127.31, 126.83, 119.79, 114.16, 45.91, 39.00, 35.69

HRMS (ESI+): *m/z*

[M + H]+ calcd for $C_{21}H_{18}NO_2$: 316.1338; found: 316.1334

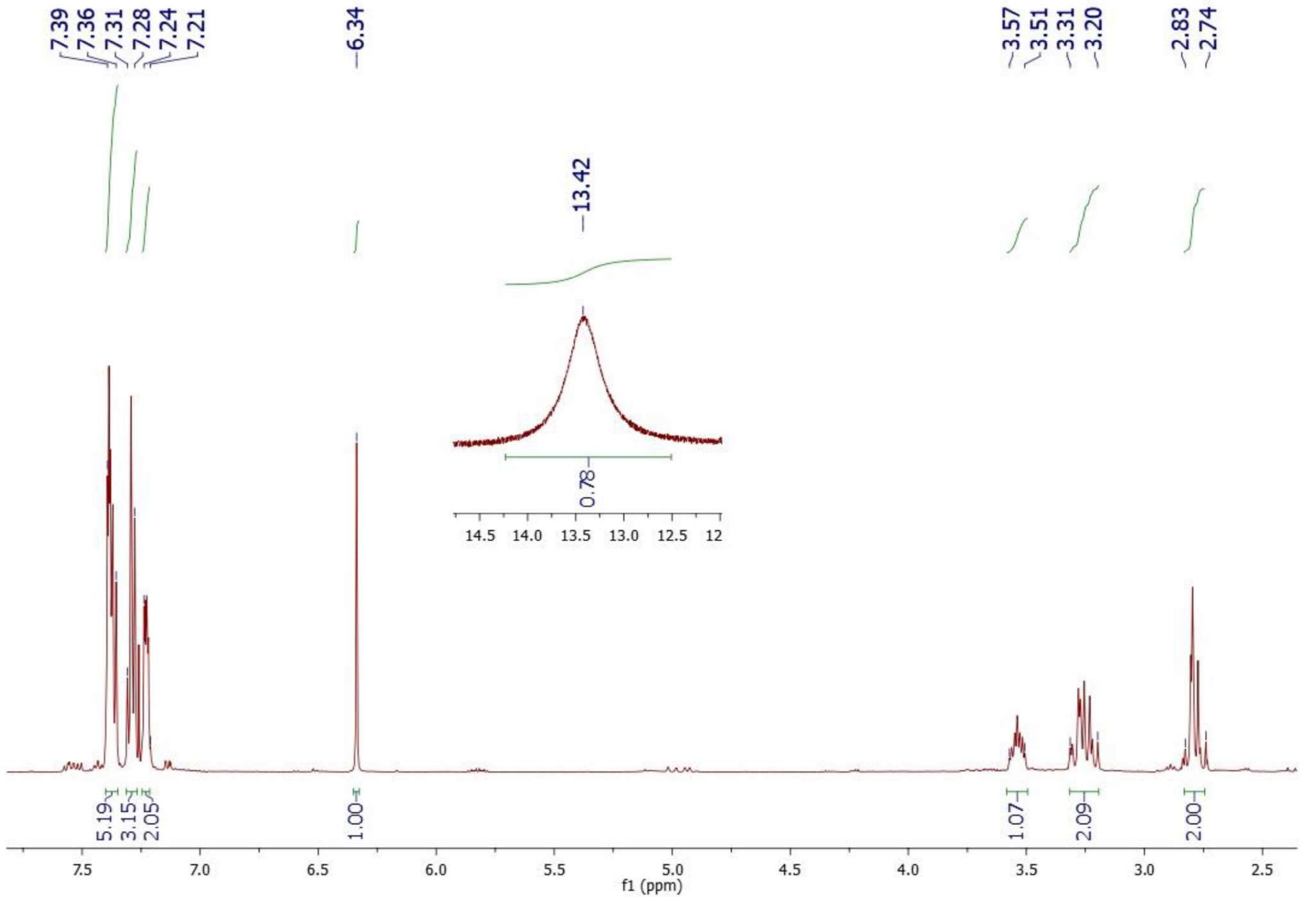

Figure S19. ^{1}H NMR of 4,7-diphenyl-1,6,7,8-tetrahydroquinoline-2,5-dione

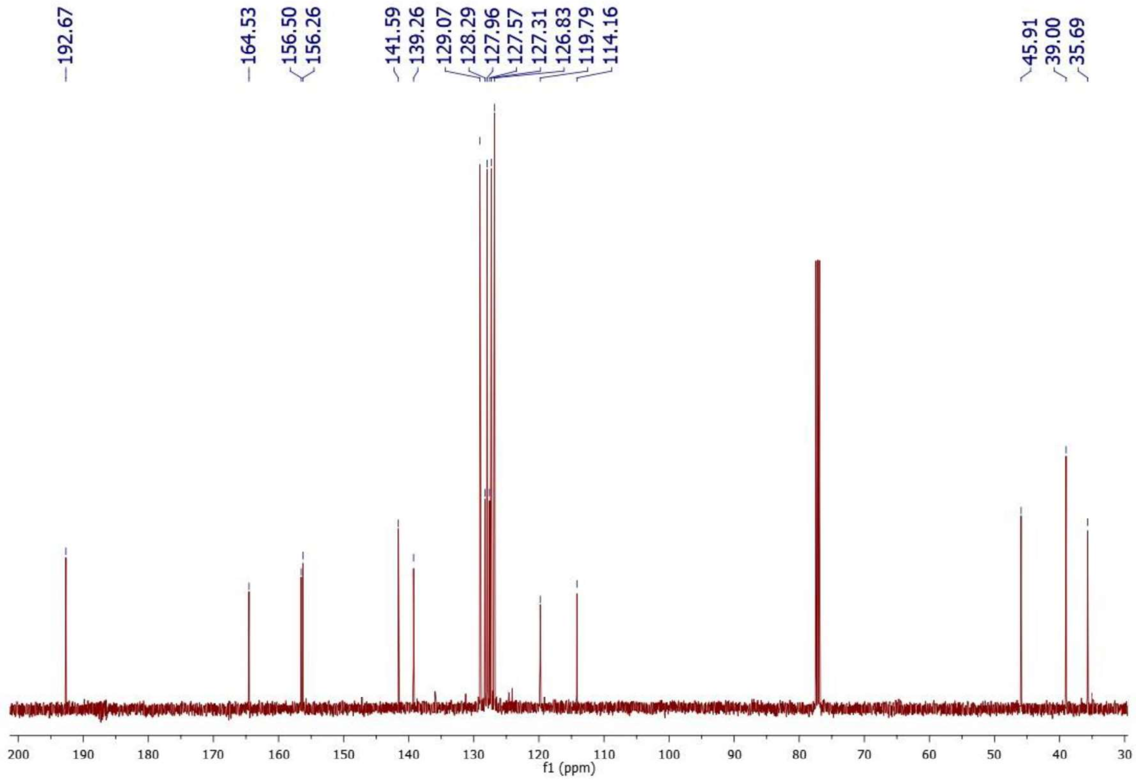

Figure S20. ^{13}C NMR of 4,7-diphenyl-1,6,7,8-tetrahydroquinoline-2,5-dione

9. 2,6-bis(2-furyl)-9-hydroxy-11,11-dimethyl-10,12-dihydro-[1,3]oxazino[2,3-j]quinoline-4,8-dione (13ga)

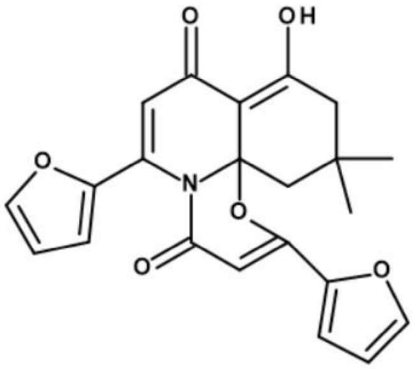

Yield: 11%,

^{1}H NMR ($CDCl_3$, 500 MHz): δ= 7.84 (d, *J* = 1 Hz, 1 H), 7.82 (d, *J* = 1 Hz, 1 H), 7.37 (d, *J* = 3.5 Hz, 1 H), 7.36 (d, *J* = 3.5 Hz, 1 H), 6.92 (s, 1 H), 6.74 (dd, *J* = 3.5 Hz, *J* = 1 Hz, 2 H), 6.70 (s, 1 H), 2.44 (s, 2 H), 2.28 (s, 2 H), 1.10 (s, 6 H)

^{13}C NMR ($CDCl_3$, 125 MHz): δ= 203.52, 178.21, 164.69, 157.10, 156.92, 153.23, 148.81, 148.26, 146.20, 145.62, 117.21, 115.19, 114.02, 114.00, 111.82, 108.50, 51.19, 42.53, 33.74, 31.82, 28.35

HRMS (ESI+): *m*/*z*

[M + Na]+ calcd for $C_{22}H_{19}NO_6Na$: 416.1110; found: 416.1108

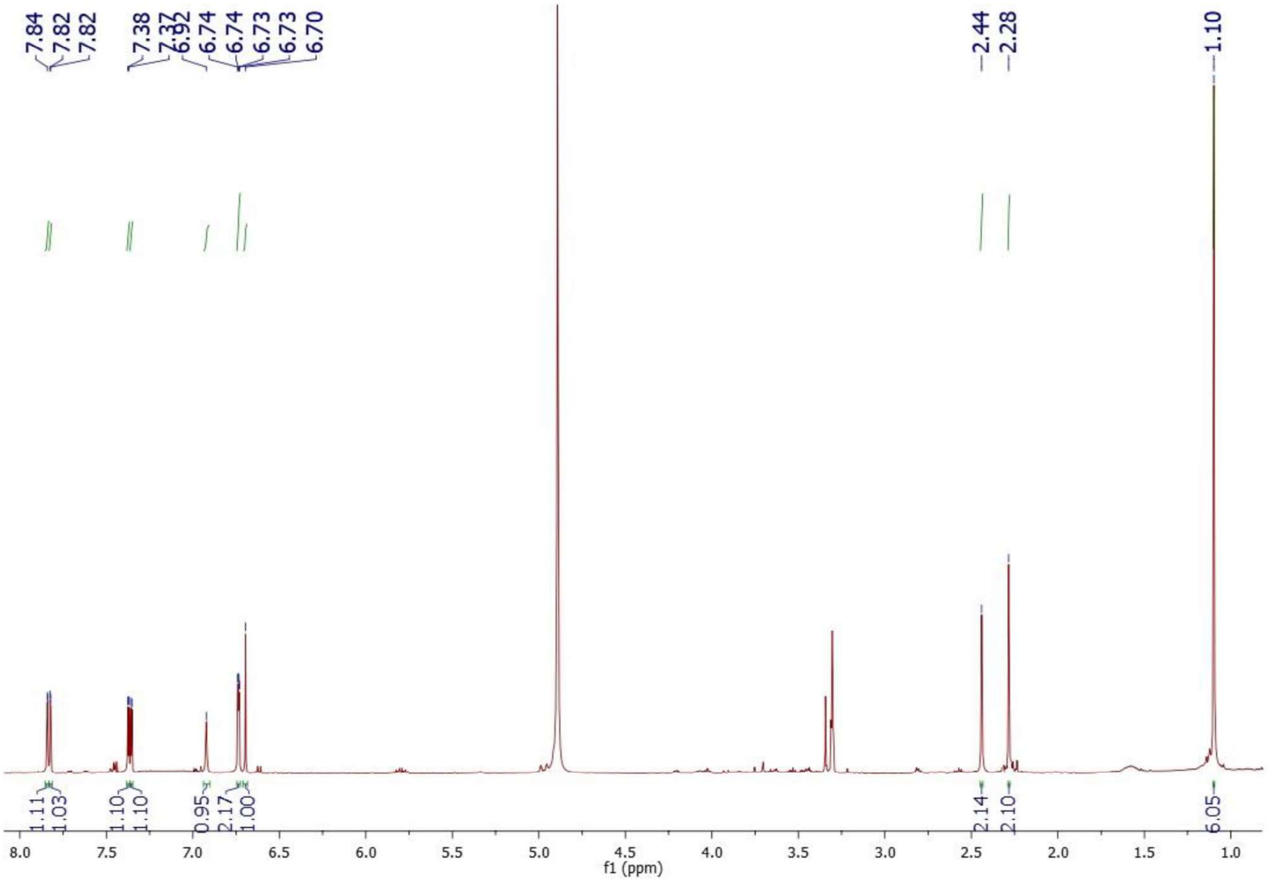

Figure S21. ^{1}H NMR of 2,6-bis(2-furyl)-9-hydroxy-11,11-dimethyl-10,12-dihydro-[1,3]oxazino[2,3-j]quinoline-4,8-dione

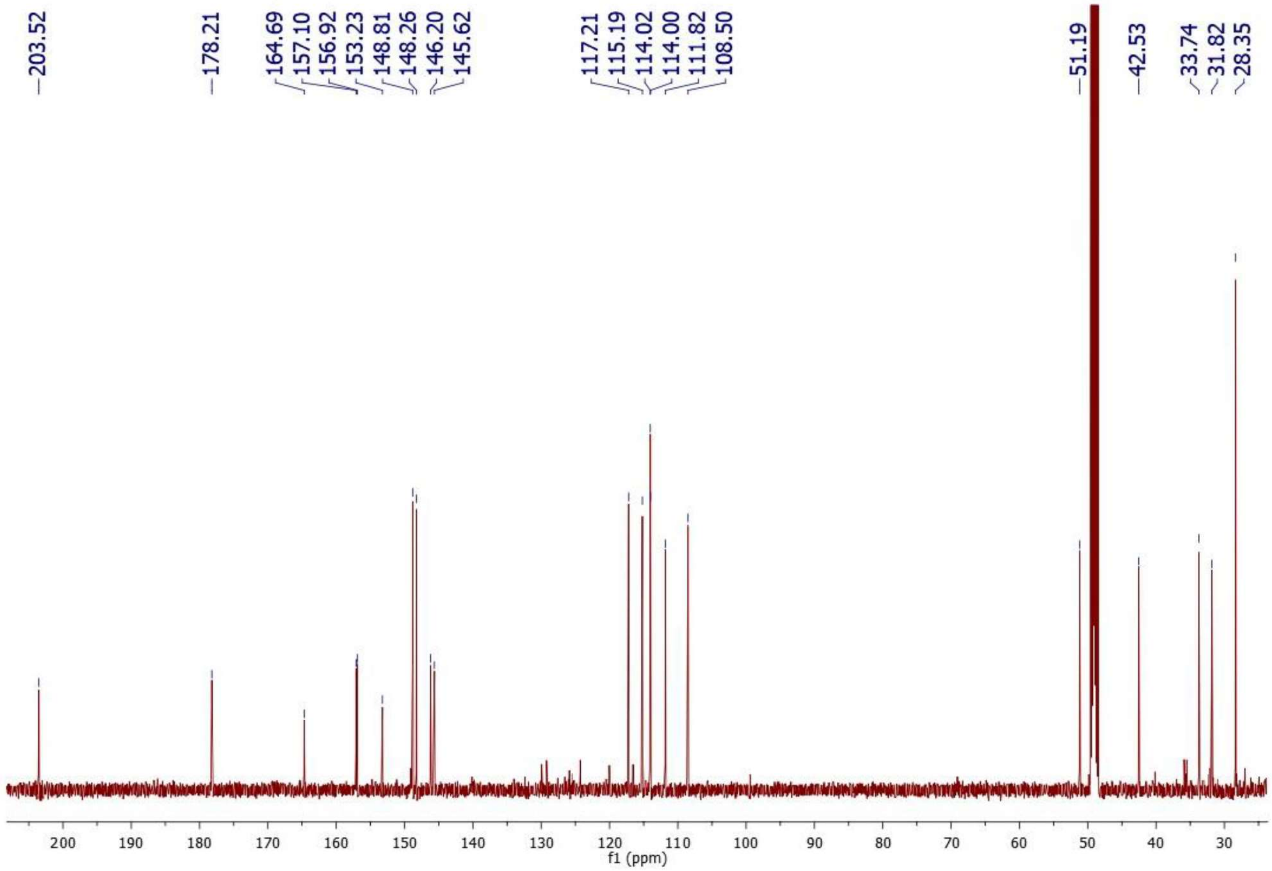

Figure S22. ^{13}C NMR of 2,6-bis(2-furyl)-9-hydroxy-11,11-dimethyl-10,12-dihydro-[1,3]oxazino[2,3-j]quinoline-4,8-dione

B. Procedure for 5-(5,5-dimethyl-3-oxo-cyclohexen-1-yl)-3-oxo-propanamide derivatives

A solution of dimedone enaminone (0.5 mmol) in 5 ml of DCE and molecular sieves were placed in a round-bottom flask with a stir bar and heated to 55°C. Then 2 mmol of acyl Meldrum's acid were added in 4 portions every 1 hour. The formation of enamide was monitored by TLC. When the spot of enaminone was no longer observed, DCE was evaporated. The final product was isolated by flash column chromatography C:M 120:1. Yields and characteristics of title compounds are collected below.

Table S1. The optimization of enamide formation

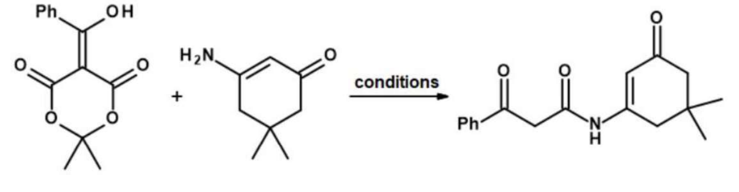

Conditions	Yield [%]
Toluene, 50 ^{o}C, 16 h, sieves	83
Toluene, 60 ^{o}C, 8 h, sieves	81
DCE, 50 ^{o}C, 16 h, sieves	94
DCE, 60 ^{o}C, 8 h, sieves	85
DCE, 55 ^{o}C, 8 h, sieves	96
DCE, 85 ^{o}C, 8 h	30

1. 5-(5,5-dimethyl-3-oxo-cyclohexen-1-yl)-3-oxo-3-phenyl-propanamide (11aa)

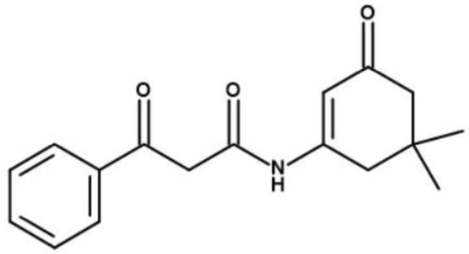

Bright yellow amorphous powder, yield: 96%

^{1}H NMR ($CDCl_3$, 500 MHz): δ= 13.88 (s, 0.22 OH), 9.96 (s, 0.7 NH), 9.95 (s, 0.3 NH), 7.96 – 7.94 (m, 1.45 H), 7.73 – 7.72 (m, 0.55 H), 7.68 – 7.65 (m, 0.7 H), 7.56 – 7. 49 (m, 2.3 H), 6.62 (s, 0.35 H), 6.60 (s, 0.65 H), 6.02 (s, 0.3 H), 4.16 (s, 1.5 H), 2.40 (s, 0.5 H), 2.32 (s, 1.5 H), 2.13 (s, 0.5 H), 2.11 (s, 1.5 H), 1.00 (s, 1.7 H), 0.98 (s, 4.3 H)

^{13}C NMR ($CDCl_3$, 125 MHz): δ= 208.19, 203.72, 176.78, 163.31, 145.50, 143.25, 138.34, 137.83, 118.87, 59.50, 57.84, 50.59, 41.73, 37.29

^{13}C NMR enole form ($CDCl_3$, 125 MHz): δ= 181.49, 142.58, 141.11, 138.49, 135.03, 119.38, 99.49, 50.76, 41.79

HRMS (ESI+): *m/z*

[M + Na]+ calcd for $C_{17}H_{20}NO_3Na$: 308.1263; found: 308.1259

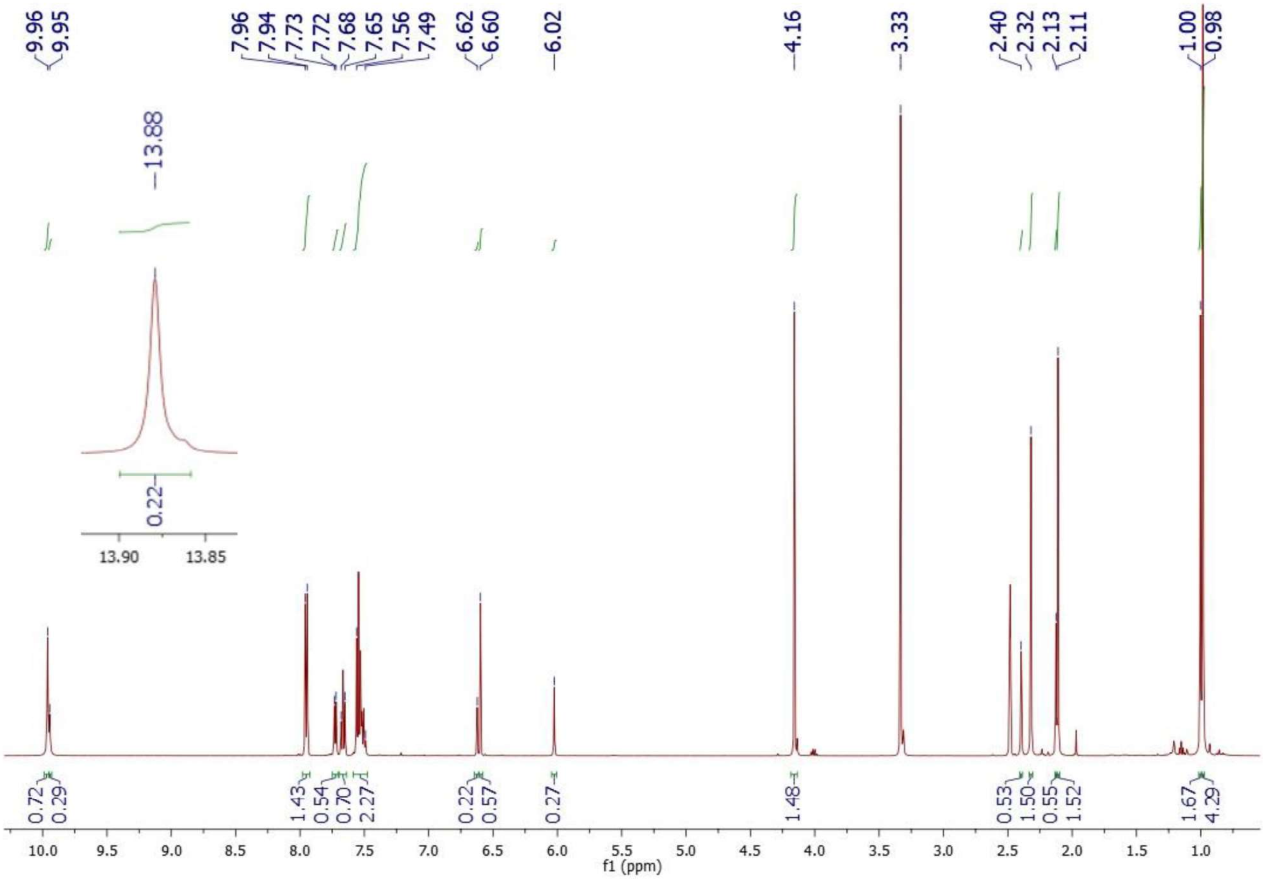

Figure S23. ^{1}H **NMR of 5-(5,5-dimethyl-3-oxo-cyclohexen-1-yl)-3-oxo-3-phenyl-propanamide**

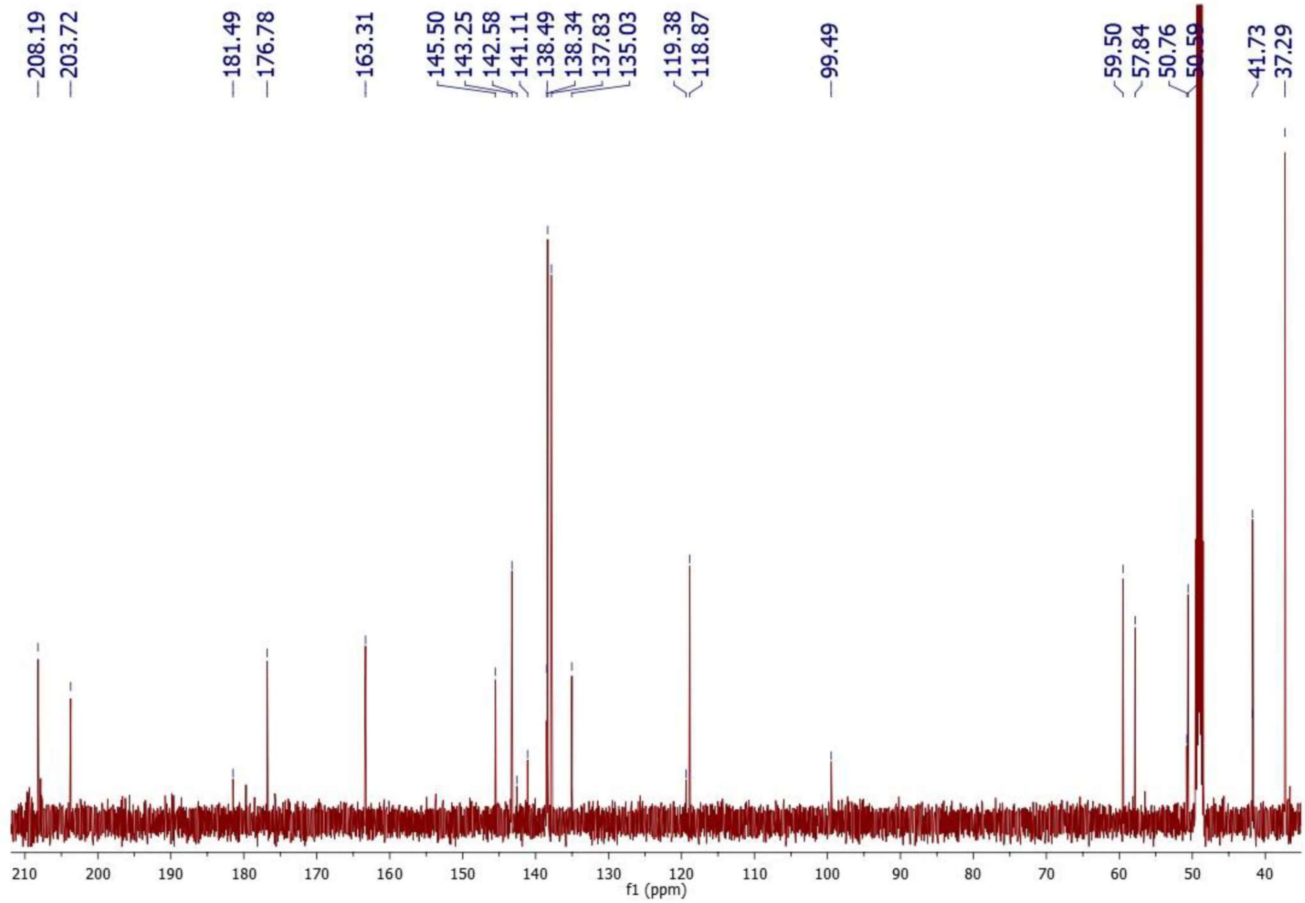

Figure S24. ^{13}C **NMR of 5-(5,5-dimethyl-3-oxo-cyclohexen-1-yl)-3-oxo-3-phenyl-propanamide**

2. 5-(5,5-dimethyl-3-oxo-cyclohexen-1-yl)-3-(4-methoxyphenyl)-3-oxo-propanamide (11ca)

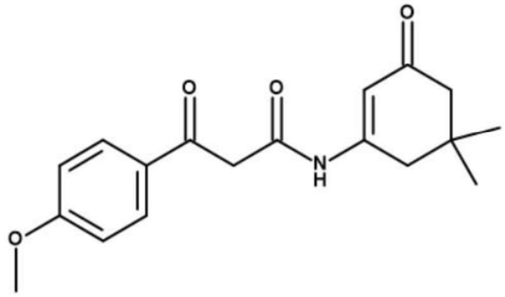

Bright yellow powder, yield: 86%

^{1}H NMR ($CDCl_3$, 500 MHz): δ= 9.56 (s, NH), 7.96 (d, *J* = 10 Hz, 2 H), 6.97 (d, *J* = 10 Hz, 2 H), 6.79 (s, 1 H), 4.04 (s, 2 H), 3.89 (s, 3 H), 2.44 (s, 2 H), 2.23 (s, 2 H), 1.09 (s, 6 H)

^{13}C NMR ($CDCl_3$, 125 MHz): δ= 200.51, 194.02, 165.43, 164.71, 131.09, 128.65, 114.23, 111.32, 55.67, 50.45, 45.24, 42.50, 32.79, 28.24

HRMS (ESI+): *m/z*

[M + H]+ calcd for $C_{18}H_{22}NO_4$: 316.1549; found: 316.1545

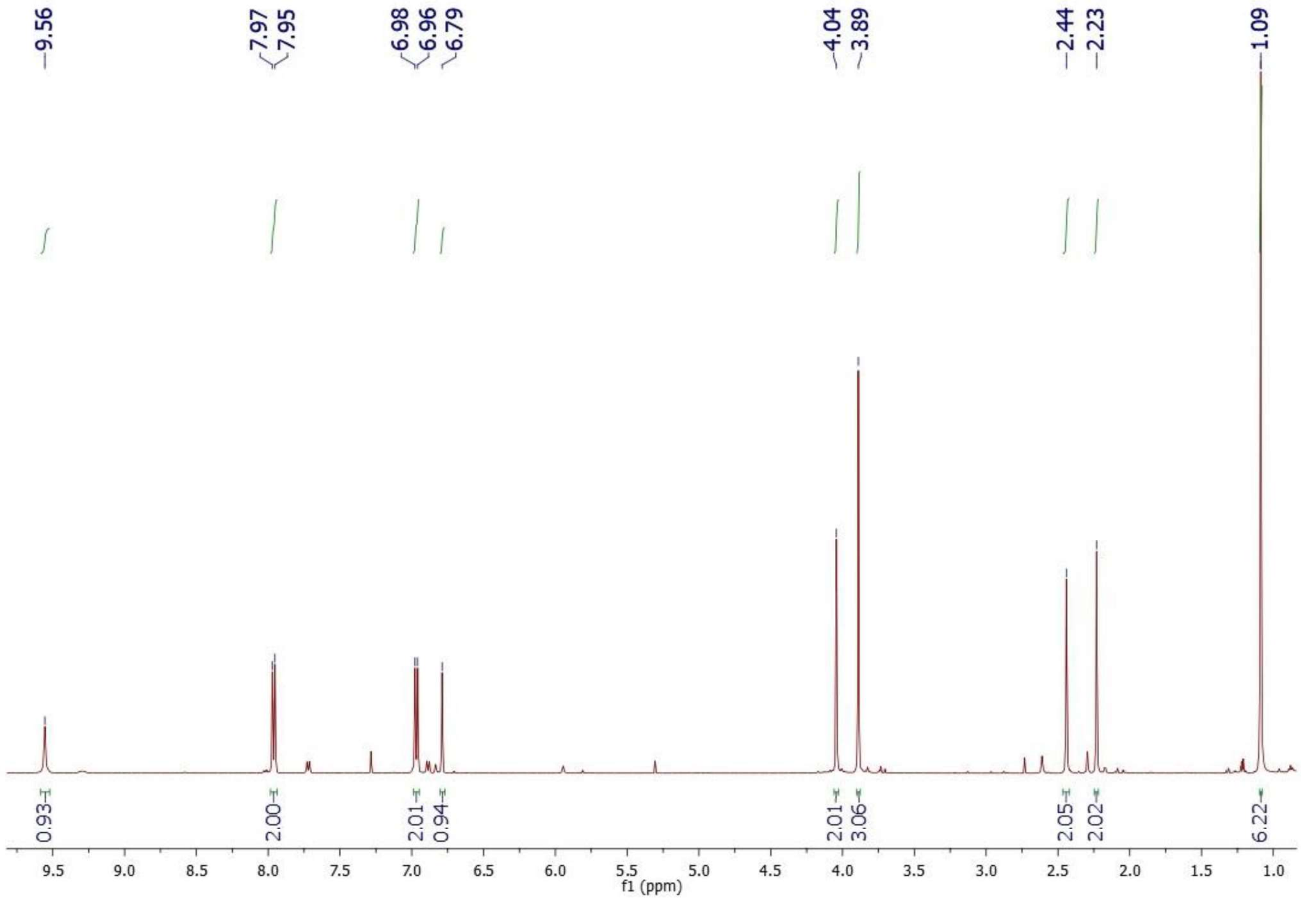

Figure S25. ^{1}H NMR of 5-(5,5-dimethyl-3-oxo-cyclohexen-1-yl)-3-(4-methoxyphenyl)-3-oxo-propanamide

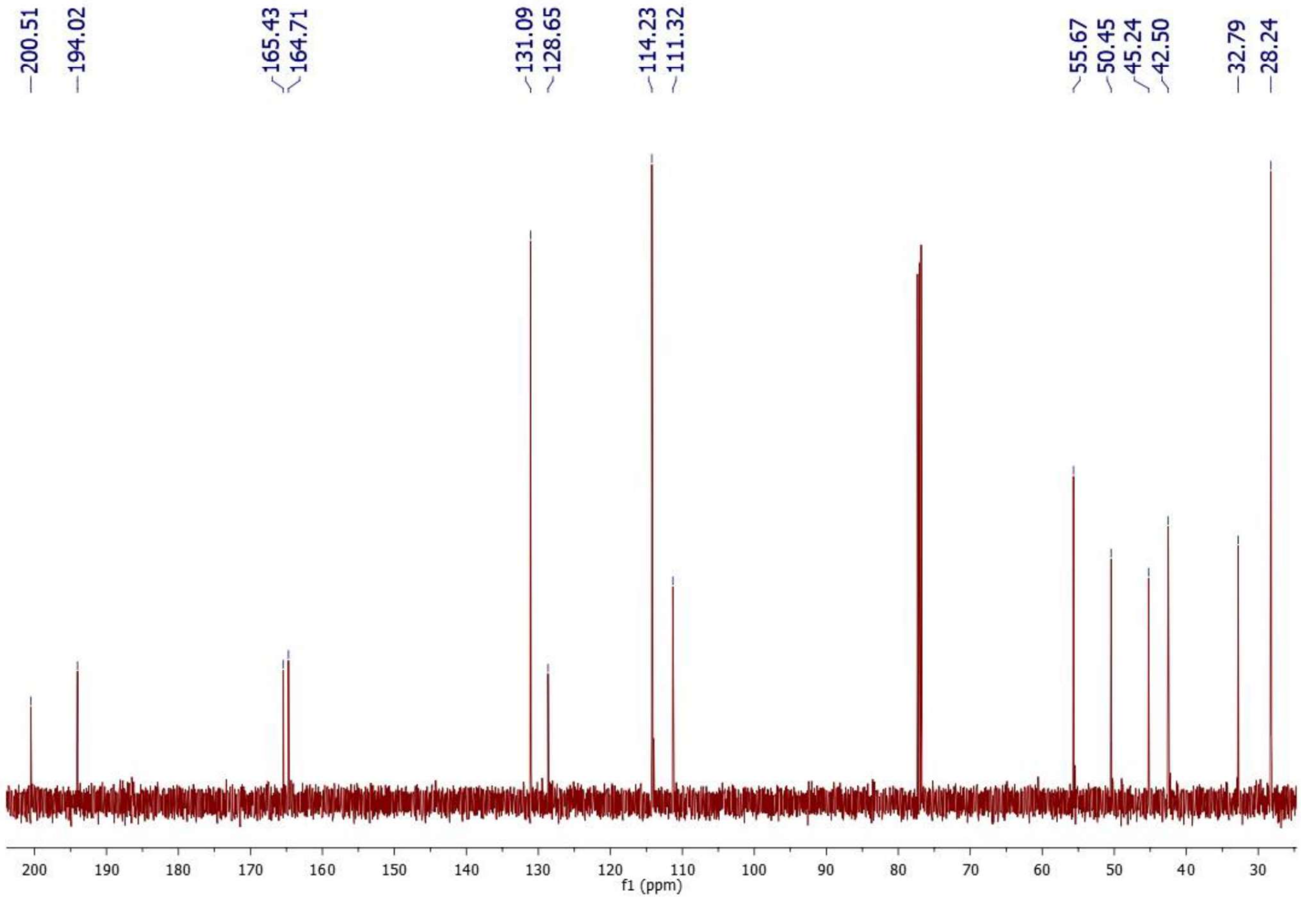

Figure S26. ^{1}H NMR of 5-(5,5-dimethyl-3-oxo-cyclohexen-1-yl)-3-(4-methoxyphenyl)-3-oxo-propanamide

3. 5-(5,5-dimethyl-3-oxo-cyclohexen-1-yl)-3-(2-furyl)-3-oxo-propanamide (11ga)

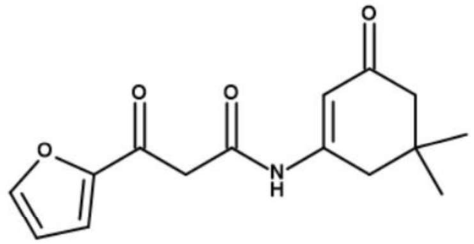

White amorphous powder, yield: 81%

^{1}H NMR ($CDCl_3$, 500 MHz): δ= 9.29 (s, NH), 7.67 (d, *J* = 1 Hz, 1 H), 7.36 (d, *J* = 3.5 Hz, 1 H), 6.74 (s, 1 H), 6.6 (dd, *J* = 3.5 Hz, *J* = 1 Hz, 1 H), 3.95 (s, 2 H), 2.41 (s, 2 H), 2.21 (s, 2 H), 1.06 (s, 6 H)

^{13}C NMR ($CDCl_3$, 125 MHz): δ= 200.56, 183.53, 164.59, 151.55, 148.38, 120.30, 113.29, 111.48, 50.54, 45.51, 42.55, 32.89, 28.31

HRMS (ESI+): *m/z*

[M + H]+ calcd for $C_{15}H_{18}NO_4$: 276.1236; found: 276.1230

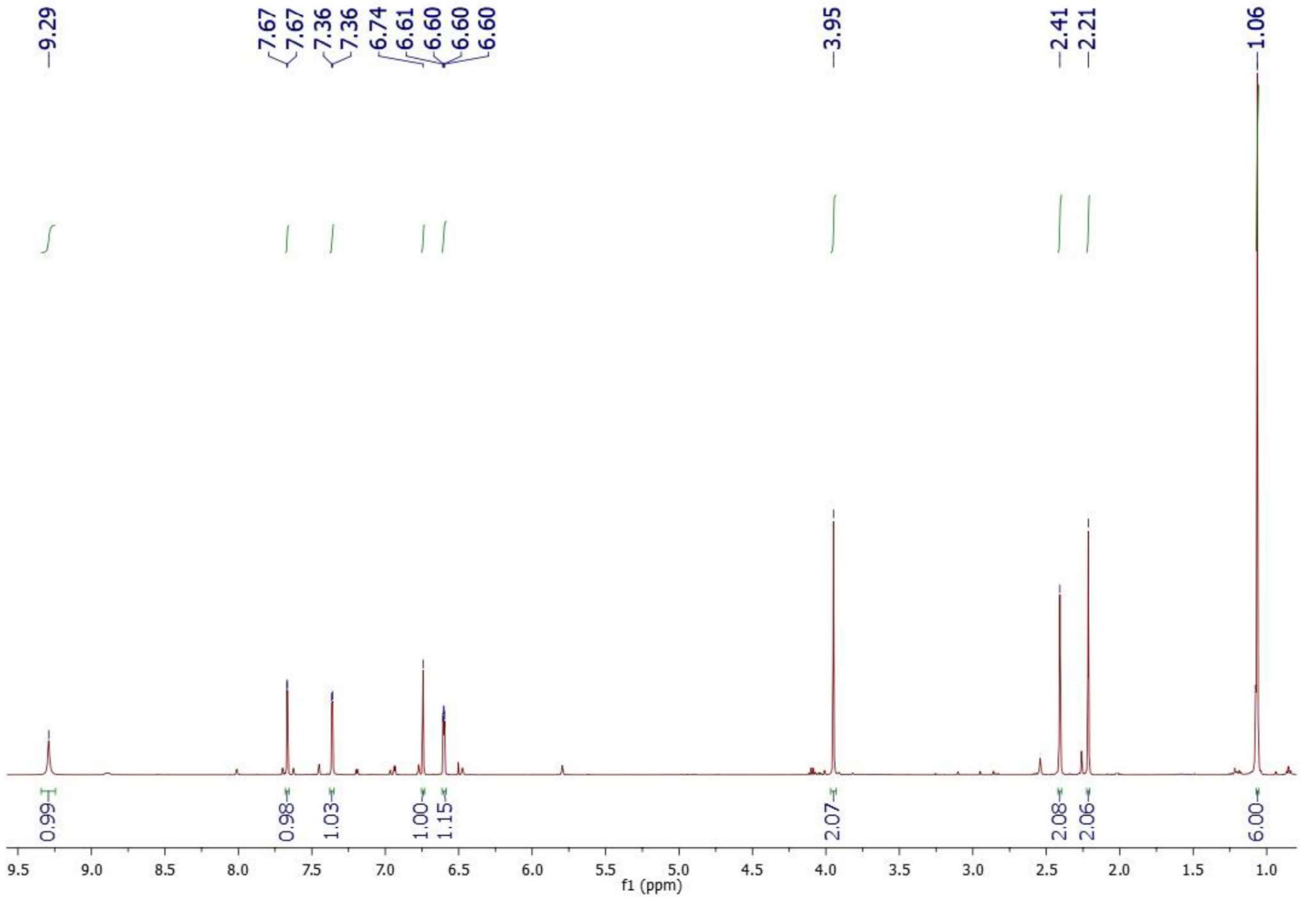

Figure S27. ^{1}H NMR of 5-(5,5-dimethyl-3-oxo-cyclohexen-1-yl)-3-(2-furyl)-3-oxo-propanamide

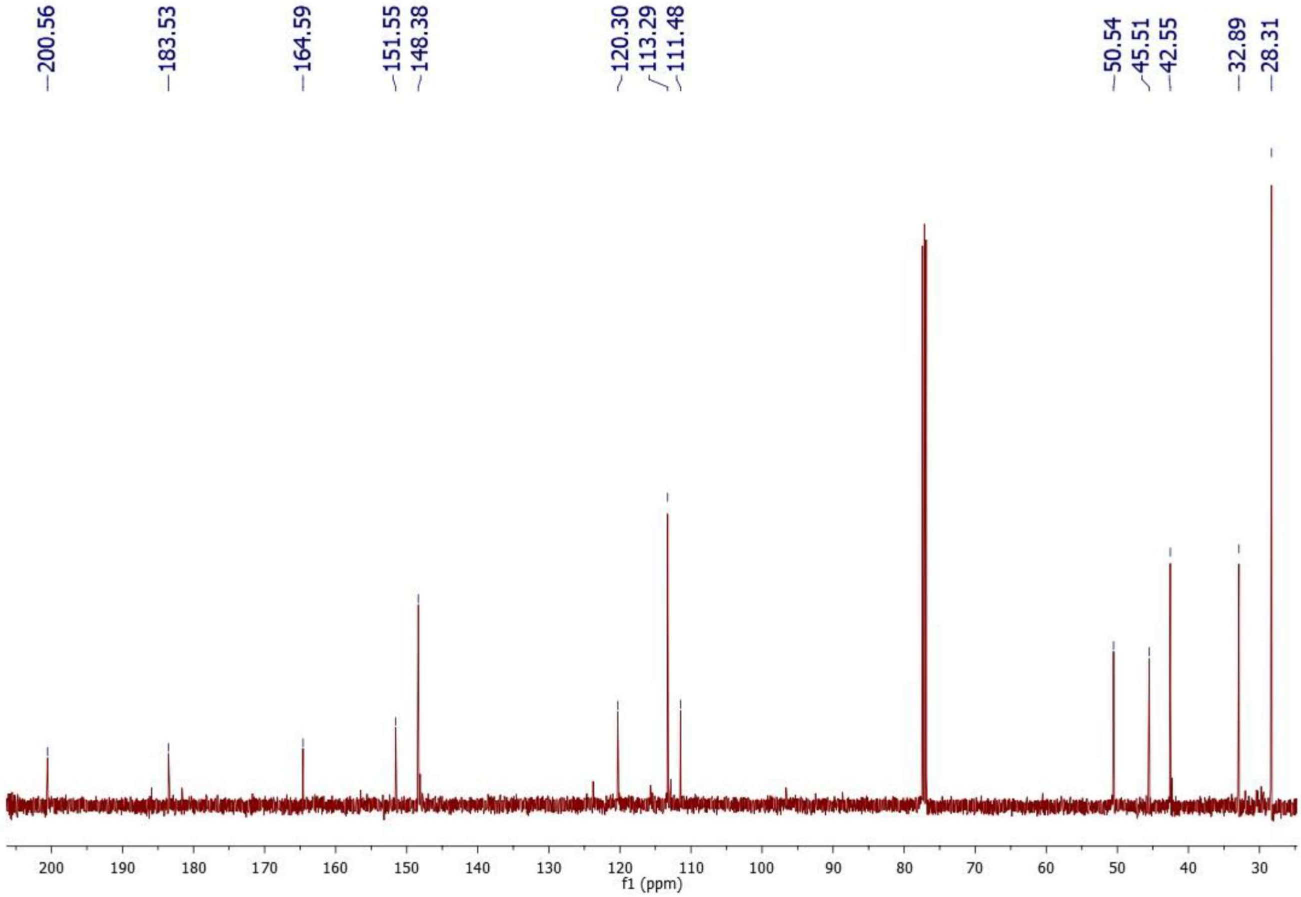

Figure S28. ^{13}C NMR of 5-(5,5-dimethyl-3-oxo-cyclohexen-1-yl)-3-(2-furyl)-3-oxo-propanamide

REFERANCES

[1] Khopade, T. M.; Warghude, P. K.; Mete, T. B.; Bhat, R. G. Acyl/aroyl Meldrum's acid as an enol surrogate for the direct organocatalytic synthesis of α,β-unsaturated ketones. *Tetrahedron Lett*. **2019**, 60, 197-200.

[2] Emtenäs, H.; Alderin, L.; Almqvist, F. An enantioselective ketene-imine cycloaddition method for synthesis of substituted ring-fused 2-pyridinones. *J. Org. Chem.* **2001**, *66,* 6756-6761.

[3] R, C.; Pise, A.; Shah, S. K.; D,R.; V, S.; Tiwari, K. N. Copper-catalyzed thiolation of terminal alkynes employing thiocyanate as the sulfur source leading to enaminone-based alkynyl sulfides under ambient conditions. *Org. Lett.* **2020**, 22, 6557–6561.
